# HDAC inhibition potentiates immunotherapy in triple negative breast cancer

**DOI:** 10.18632/oncotarget.23169

**Published:** 2017-12-12

**Authors:** Manuela Terranova-Barberio, Scott Thomas, Niwa Ali, Nela Pawlowska, Jeenah Park, Gregor Krings, Michael D. Rosenblum, Alfredo Budillon, Pamela N. Munster

**Affiliations:** ^1^ Division of Hematology and Oncology, University of California, San Francisco, California, USA; ^2^ Department of Dermatology, University of California, San Francisco, California, USA; ^3^ Division of Pathology, University of California, San Francisco, California, USA; ^4^ Experimental Pharmacology Unit, Istituto Nazionale Tumori Fondazione G. Pascale - IRCCS, Naples, Italy

**Keywords:** HDAC inhibitor, immunotherapy, checkpoint inhibitor, triple negative breast cancer, epigenetics modulators

## Abstract

Triple-negative breast cancer (TNBC) represents a more aggressive and difficult subtype of breast cancer where responses to chemotherapy occur, but toxicity is significant and resistance often follows. Immunotherapy has shown promising results in various types of cancer, including breast cancer. Here, we investigated a new combination strategy where histone deacetylase inhibitors (HDACi) are applied with immune checkpoint inhibitors to improve immunotherapy responses in TNBC.

Testing different epigenetic modifiers, we focused on the mechanisms underlying HDACi as priming modulators of immunotherapy. Tumor cells were co-cultured with human peripheral blood mononuclear cells (PBMCs) and flow cytometric immunophenotyping was performed to define the role of epigenetic priming in promoting tumor antigen presentation and immune cell activation. We found that HDACi up-regulate PD-L1 mRNA and protein expression in a time-dependent manner in TNBC cells, but not in hormone responsive cells. Focusing on TNBC, HDACi up-regulated PD-L1 and HLA-DR on tumor cells when co-cultured with PBMCs and down-regulated CD4^+^ Foxp3^+^ Treg *in vitro*. HDACi significantly enhanced the *in vivo* response to PD-1/CTLA-4 blockade in the triple-negative 4T1 breast cancer mouse model, the only currently available experimental system with functional resemblance to human TNBC. This resulted in a significant decrease in tumor growth and increased survival, associated with increased T cell tumor infiltration and a reduction in CD4^+^ Foxp3^+^ T cells in the tumor microenvironment. Overall, our results suggest a novel role for HDAC inhibition in combination with immune checkpoint inhibitors and identify a promising therapeutic strategy, supporting its further clinical evaluation for TNBC treatment.

## INTRODUCTION

Breast cancer is one of the most common diseases, second only to lung cancer as the leading cause of cancer death in women, accounting for 30% of new diagnoses [1]. Triple-negative breast cancer (TNBC) is a heterogeneous subtype of breast cancer with poor prognosis and high risk of relapse. Despite initial response to therapy, resistance develops in the majority of patients [2]. Current approved therapeutic options for TNBC are limited to anthracyclines, taxanes and anti-metabolites [3].

Histone deacetylase inhibitors (HDACi) represent a new class of anticancer agents that induce a wide range of transient gene expression alterations, without implicating permanent changes in DNA sequence [4, 5]. The cellular response to HDACi is complex and involves the regulation of histone and non-histone proteins by modifying their post-translational acetylation, thus playing a critical role in various cancer pathways. In addition to their effects on cancer signaling, HDACi have distinct immune modulatory functions, including modulation of regulatory T cells (Treg), Foxp3 expression and changes in tumor-infiltrating lymphocytes (TILs) composition [6, 7]. Emerging data suggest that epigenetic modulation is important for controlling T cells infiltration, differentiation, and PD-L1 expression [7-9].

In this context, the introduction of immunotherapy to cancer treatment is providing significant clinical benefit against immunogenic tumors, such as melanoma [7, 10] and has created high hopes for TNBC treatment. Antigen-specific immune responses are complex and highly regulated, involving stimulatory/inhibitory coupling of receptors and ligands that can be specifically directed against cancer cells [11]. The PD-1/PD-L1 pathway is a key inhibitory pathway [12, 13]. When activated, it regulates cytotoxic T cells activity and helps to avoid autoimmunity and maintain immune system homeostasis [14, 15]. In the tumor microenvironment, cancer exploits this pathway to suppress immune response and inhibit cytotoxic T cells activity.

In breast cancer, PD-L1 expression is primarily found in Her2^+^ and TNBC. Increased PD-L1 expression correlated with higher TILs, which are in turn associated with a decreased rate of recurrence, better response, better survival and stronger cytotoxic immune response [16, 17]. PD-L1 over-expression in basal breast tumors has also been associated with increased expression of immune response related genes, with pathways that are involved in T cells activation, differentiation and expression of anti-tumor cytokines and with better response to anthracycline-based neoadjuvant chemotherapy [18, 19]. Thus, inhibition of PD-1/PD-L1 interactions may prevent T cells suppression and reactivate immune-surveillance.

Although HDAC inhibition has been reported to increase PD-1 blockade efficacy in melanoma and lung adenocarcinoma [9, 20], its potential role in breast cancer as immune modulator and the mechanism promoting such strategy has not been investigated. Additionally, PD-L1 expression as a predictive marker for checkpoint inhibitors response has not been clearly established in breast cancer. Compared to lung cancer and melanoma, breast cancer appears less immunogenic, which may explain the relatively low response rate to single agent pembrolizumab [21]. Given the limited response to immunotherapy, we explored the potential of epigenetic modulation with HDACi to boost response to immune checkpoint inhibitors in both estrogen receptor-negative (ER^-^) and ER^+^ breast cancer. We found that HDAC inhibition leads to PD-L1 up-regulation on tumor cells and pre-requisite cell surface expression. Only seen in ER^-^ cell lines, PD-L1 up-regulation was associated with increased HLA-DR tumor cells expression, CD4^+^ Foxp3^+^ CTLA-4^high^ Treg down-regulation *in vitro*, increased T cells tumor infiltration, longer survival and tumor growth inhibition *in vivo*. Taken together, our data suggest that HDACi potentiate immune checkpoint inhibitor blockade in TNBC.

## RESULTS

### HDACi up-regulate PD-L1

Multiple human breast cancer cell lines, which represent the molecular diversity of breast cancer, were tested for basal PD-L1 expression (Supplementary Figure 1A).

Expression varied significantly. Triple-negative MDA-MB231 cells exhibited the highest basal level of PD-L1 protein and mRNA, while MCF-7 cells (ER^+^, PR^+^ and HER2^+^) the lowest. Although SKBR3 cells (ER^-^, PR^-^, HER2^+^) expressed more PD-L1 mRNA than T47D cells (ER^+^, PR^+^, HER2^-^), protein expression was comparable (Supplementary Figure 1A and 1B).

**Figure 1:**
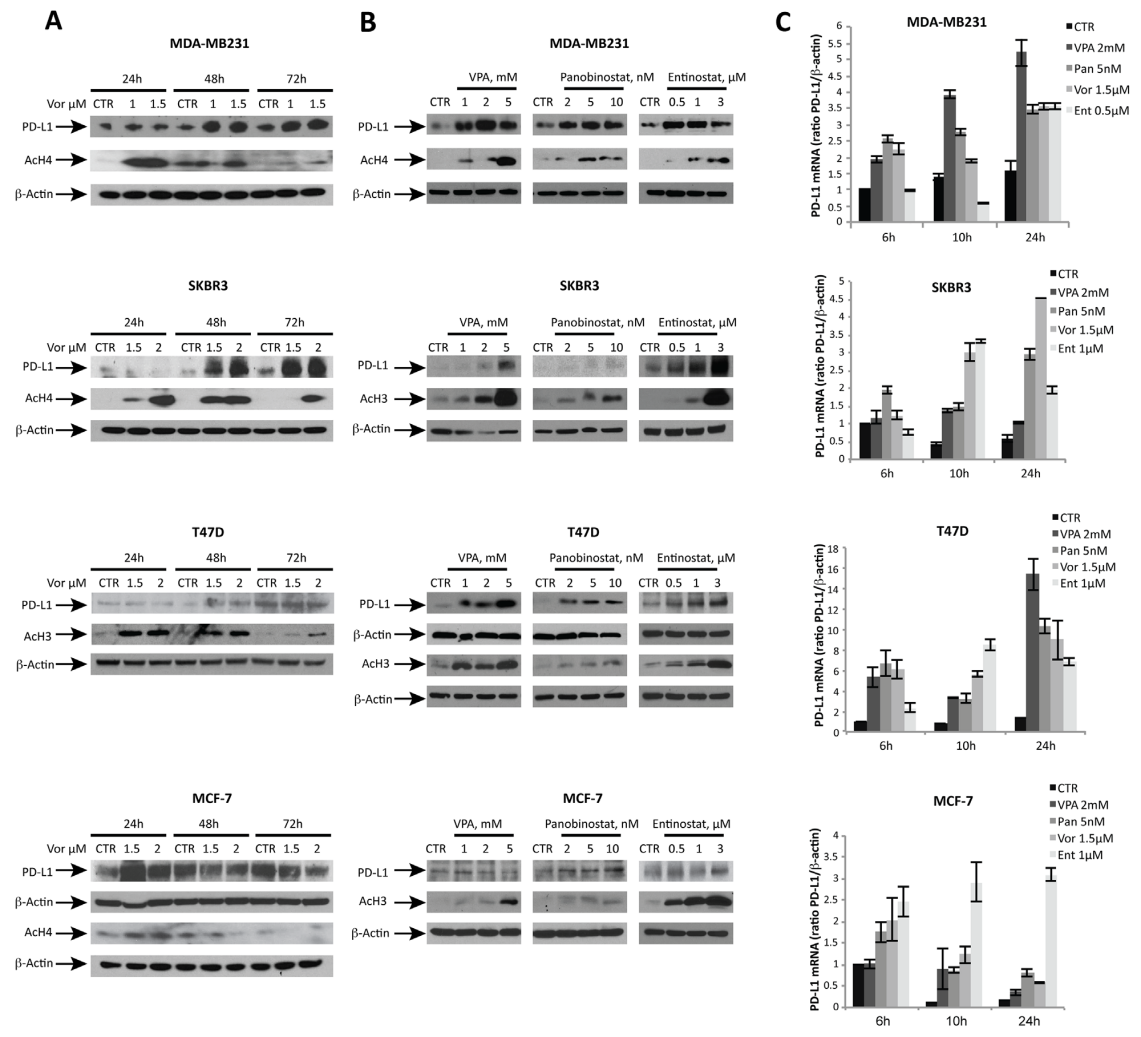
HDACi modulate PD-L1 expression in ER^-^ breast cancer cell lines in a dose- and time-dependent manner **(A)** PD-L1, acetyl-H3 (AcH3) and acetyl-H4 (AcH4) protein expression was determined by western blot in MDA-MB231, SKBR3, T47D and MCF-7 cells untreated or treated for 24, 48 and 72 hours with increasing doses of vorinostat. **(B)** PD-L1, AcH3, AcH4 protein expression was evaluated in MDA-MB231, SKBR3, T47D and MCF-7 cells untreated or treated for 48 hours with different HDACi: valproic acid (VPA), panobinostat and entinostat. **(C)** PD-L1 mRNA expression was evaluated by qReal-Time PCR in MDA-MB231, SKBR3, T47D and MCF-7 cells untreated or treated with various HDACi for 6, 10 and 24 hours. β-actin was used as protein loading control in western blot or housekeeping control gene to normalize qReal-Time PCR reactions.

To investigate PD-L1 modulation by HDACs, several breast cancer cell lines were treated with increasing doses of vorinostat. A dose-dependent up-regulation of PD-L1 protein expression was detected after 48 hours of treatment, which differed significantly between ER^-^ versus ER^+^ breast cancer cells (Figure 1A). These effects were observed with class non-specific HDACi (vorinostat and panobinostat), but also with class specific HDACi such as valproic acid (VPA) and entinostat (Figure 1B). A relative quantification between western blot experiments is shown in Supplementary Figure 2. Moreover a time-dependent increase in PD-L1 mRNA was observed with various HDACi, comparable in trend to protein modulation (Figure 1C).

### HDACi up-regulate cell surface PD-L1 expression inTNBC cells

**Figure 2:**
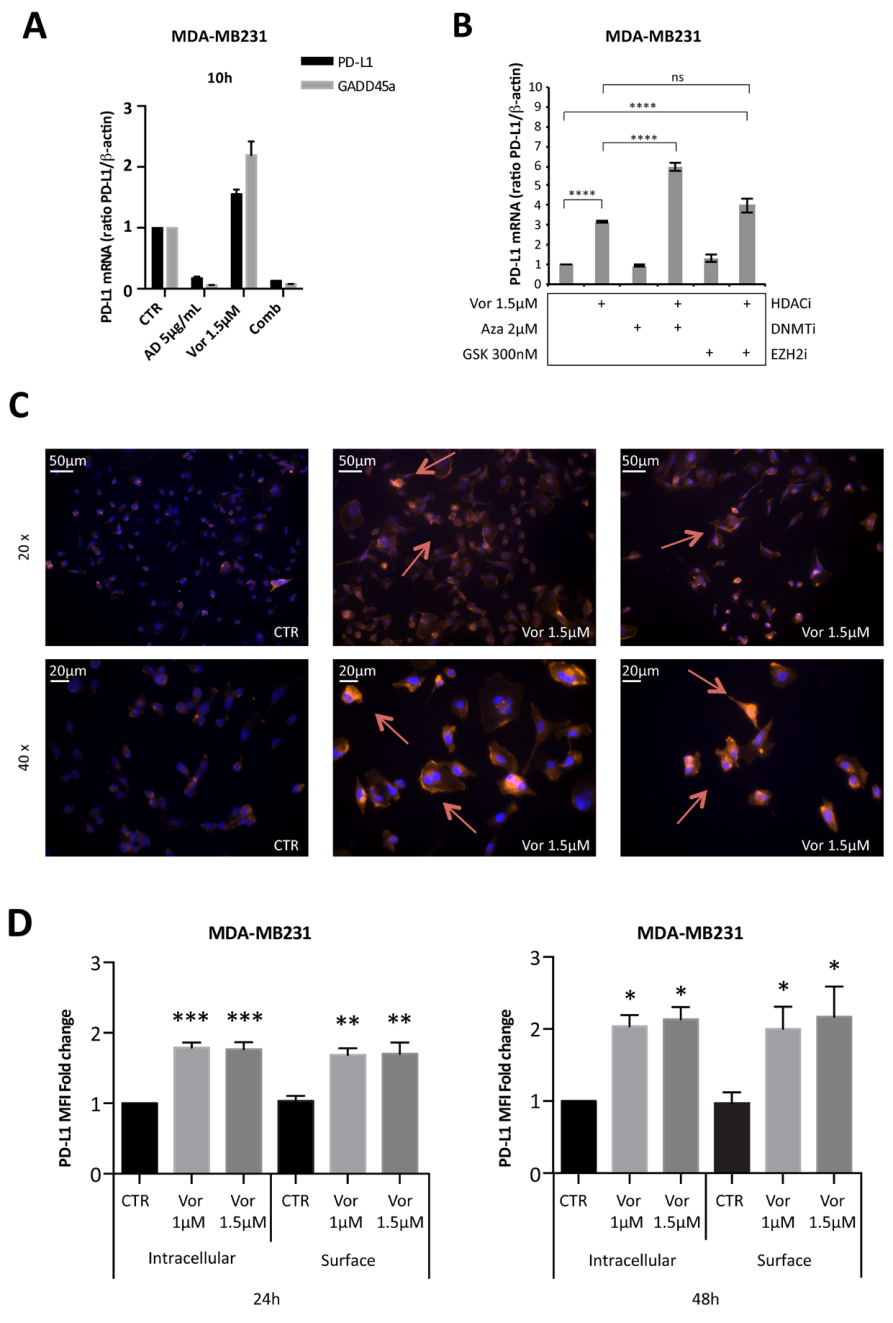
Vorinostat induces PD-L1 expression in breast cancer cell lines directly at a transcriptional level and this translates to increased PD-L1 expression on the cell surface **(A)** MDA-MB231 cells were untreated or treated with vorinostat (1.5μM) and/or actinomycin D (AD 5μg/mL) for 10 hours. The expression levels of PD-L1 and GADD45a mRNA were determined by qReal-Time PCR. **(B)** MDA-MB231 were untreated or treated with vorinostat (1.5μM) alone or in combination with azacitidine (Aza 2μM) or GSK126 (GSK 300nM). PD-L1 mRNA expression was evaluated by qReal-Time PCR with β-actin used as housekeeping control gene. **(C)** MDA-MB231 cells untreated or treated with vorinostat (1.5μM) for 48 hours, were fixed, stained for PD-L1 (red) and DAPI for nuclei (blue) and observed by microscope. Representative images show PD-L1^+^ cells with 20x or 40x magnification. **(D)** MDA-MB231 untreated or treated with increasing doses of vorinostat for 24 and 48 hours were collected for flow cytometry analysis: cells were stained for PD-L1 before or after fixation/permeabilization steps to distinguish between surface and intracellular markers staining, respectively. Flow cytometric quantification of PD-L1 MFI in live tumor cells is shown (expressed as fold change relative to the control). Statistical comparisons are relative to respective intracellular or surface control. Statistical significance is indicated by p-values as ^*^ P ≤ 0.05; ^**^ P ≤ 0.01; ^***^ P ≤ 0.001; ^****^ P ≤ 0.0001 and ns: non significant. Data are presented as the mean ± SD for (A) and (B) and ± SEM for (D).

We then investigated the mechanism of HDACi-mediated induction of PD-L1 and its cellular localization in triple-negative MDA-MB231 cells, characterized by higher basal expression and a significant increase of PD-L1 after HDACi treatment. Like for GADD45a, a gene up-regulated by HDACi at the transcriptional level [22], the HDACi-mediated increase of PD-L1 mRNA was blocked by concurrent treatment with the transcriptional inhibitor actinomycin-D, suggesting a transcriptional effect of HDACi (Figure 2A).

HDACi effect on PD-L1 expression was compared to other epigenetic drugs including azacitidine, a DNA methyltransferase inhibitor (DNMTi) and GSK126, a EZH2 methyltransferase inhibitor. Combination treatment of vorinostat and azacitidine resulted in a greater up-regulation of PD-L1 mRNA expression (Figure 2B). The addition of GSK126 enhanced the effect of vorinostat, but to a much lower extent compared to the DNMTi (Figure 2B).

To identify HDAC isoforms involved in PD-L1 modulation, we treated MDA-MB231 cells with HDAC1, HDAC2, HDAC3, or HDAC6 siRNAs. Neither HDAC1, HDAC2, HDAC3 single silencing, a combination of the three, nor HDAC6 silencing resulted in increased PD-L1 transcript in MDA-MB231 cells (Supplementary Figure 3A). Although, a clear interpretation of these results is complicated as HDACs silencing involves non-specific and compensatory effects that could affect the results obtained (Supplementary Figure 3B and previous reports [23, 24]). This suggests that the regulation of PD-L1 in MDA-MB231 cells involves multiple HDAC enzymes and illustrates their redundant activity.

As a cell surface marker, PD-L1 is transported to the cell membrane to carry out its function [25]. To test whether HDACi-mediated PD-L1 modulation resulted in increased cell surface expression, cells were evaluated by immunofluorescence microscopy. Treatment of MDA-MB231 cells with vorinostat (1.5 μM) for 48 hours increased the expression of PD-L1 protein (Figure 2C). In contrast, in MCF-7 cells, surface PD-L1 was not detected before or after vorinostat treatment (Supplementary Figure 4A), consistent with the modest basal and HDACi-mediated expression, previously shown (Figure 1). To confirm the increased PD-L1 expression on the cell membrane, cells were treated with vorinostat, stained for PD-L1 antibody before or after cell membrane permeabilization and assessed by flow cytometry for PD-L1 expression in live cells. Autofluorescence was determined by the use of fluorescence minus one (FMO) control. The mean fluorescent intensity (MFI) of PD-L1 expression was determined and, once autofluorescence MFI values were subtracted, the adjusted MFI values were graphed. Vorinostat increased PD-L1 MFI in both permeabilized and unpermeabilized cells, consistent with HDACi-mediated PD-L1 up-regulation resulting in increased cell surface expression (Figure 2D).

In its initial identification, the PD-1 gene was suggested to be associated with programmed cell death induction [26]. To confirm that the PD-L1 up-regulation we observed upon vorinostat treatment was not associated with an apoptotic effect, we compared the effect of vorinostat to the well-established apoptotic-inducing agent, epirubicin. Notably, epirubicin treatment was not associated with PD-L1 induction (Supplementary Figure 5). Importantly, the PD-L1 up-regulation we observed after vorinostat treatment, at the doses used in our study, was not associated with an apoptotic effect, as demonstrated by the absence of PARP cleavage (Supplementary Figure 5B).

### Vorinostat effect on tumor cells co-cultured with human peripheral blood mononuclear cells (PBMCs)

**Figure 3:**
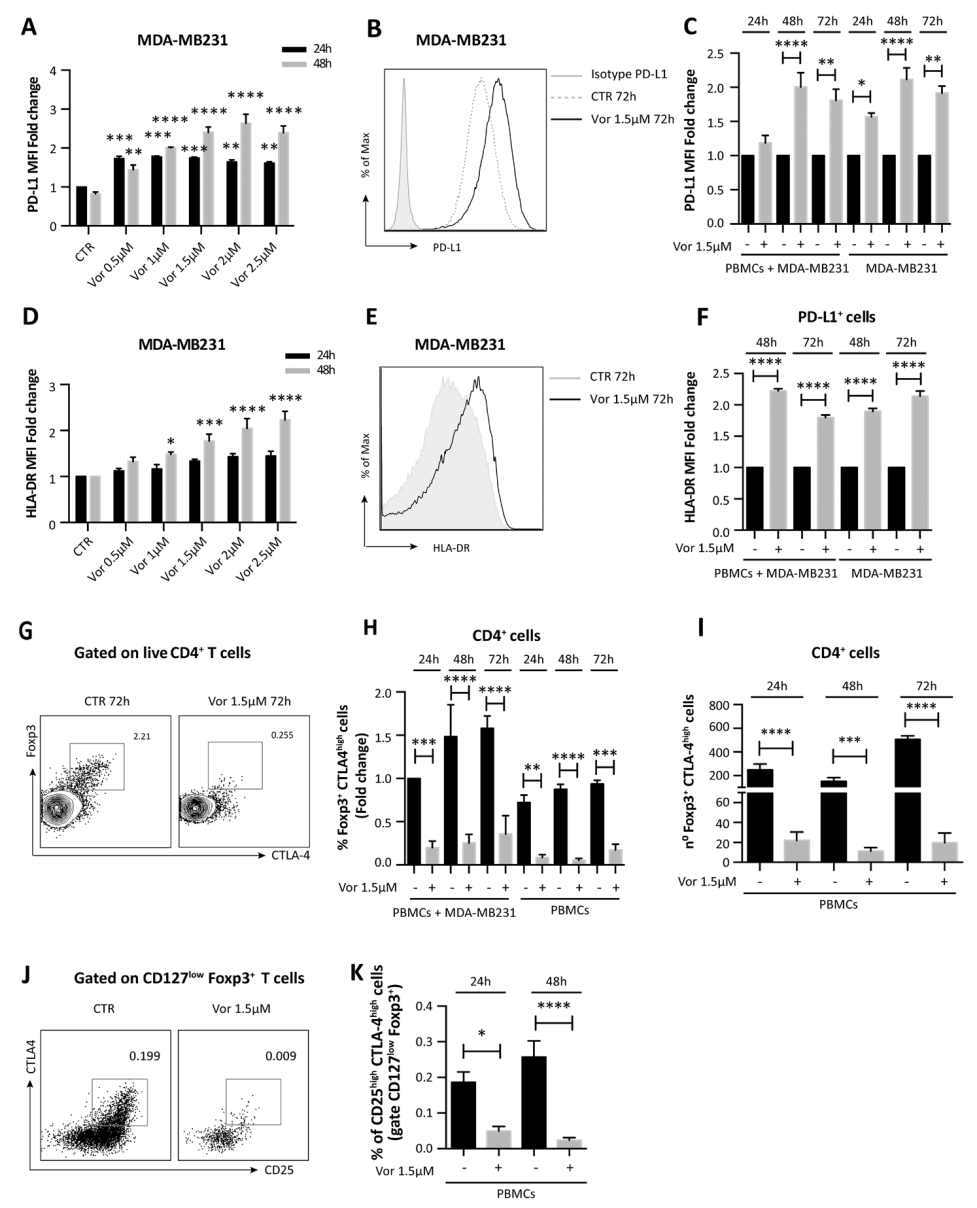
Vorinostat effect on TNBC cells and PBMCs co-cultured together **(A)** MDA-MB231 cells untreated or treated with increasing doses of vorinostat were collected 24 and 48 hours after treatment and stained for flow cytometry analysis to quantify PD-L1 expression (expressed as fold change relative to the control). Statistical comparisons are relative to respective control at 24 or 48 hours. **(B)** MDA-MB231 cells were cultured alone or in presence of PBMCs obtained from healthy donors. Cells were untreated or treated with vorinostat (1.5μM) for 24, 48 and 72 hours and then collected and stained with a comprehensive multicolor flow cytometry panel. Representative flow cytometric plot for PD-L1 expression in MDA-MB231 with or without vorinostat treatment. **(C)** Flow cytometric quantification of PD-L1 expression (expressed as fold change relative to the control) in MDA-MB231 cells alone or co-cultured with PBMCs with or without vorinostat treatment. **(D)** Flow cytometric quantification of HLA-DR expression (expressed as fold change relative to the control) in MDA-MB231 cells treated or untreated with increasing doses of vorinostat for 24 and 48 hours. Statistical comparisons are relative to respective control. **(E)** Representative flow cytometric plot for HLA-DR expression in MDA-MB231 with or without vorinostat treatment. **(F)** Flow cytometric quantification of HLA-DR expression (expressed as fold change relative to the control) in MDA-MB231 cells alone or co-cultured with PBMCs with or without vorinostat treatment. **(G)** Representative flow cytometric plots for Foxp3^+^ CTLA-4^high^ cells in PBMCs with or without vorinostat treatment. **(H)** Flow cytometric quantification (expressed as fold change relative to the control) of CD4^+^ Foxp3^+^ CTLA-4^high^ T cells in PBMCs from healthy donors alone or in presence of MDA-MB231 after 24, 48 and 72 hours of vorinostat treatment. **(I)** Flow cytometric quantification of the number of CD4^+^ Foxp3^+^ CTLA-4^high^ T cells in PBMCs with or without vorinostat treatment for 24, 48 and 72 hours. **(J)** Representative flow cytometric plots for CD25/CTLA-4 co-expression in live CD4^+^, CD127^low^, Foxp3^+^ PBMCs with or without vorinostat treatment. **(K)** Flow cytometric quantification of CD25^high^ CTLA-4^high^ Treg in PBMCs of healthy donors with or without vorinostat treatment for 24 and 48 hours. Plots in (J) and (K) are pre-gated on live CD4^+^ CD127^low^ Foxp3^+^ cells. Data are presented as the mean ± SEM. Statistical significance is indicated by p-values as ^*^ P ≤ 0.05; ^**^ P ≤ 0.01; ^***^ P ≤ 0.001; ^****^ P ≤ 0.0001.

We further confirmed that PD-L1 expression was enhanced with vorinostat treatment in a dose- and time-dependent manner (Figure 3A).

To play an active role in tumor escape, PD-1, expressed by TILs, must engage with PD-L1, on tumor cells surface. Since the interaction between these two cell types in the tumor microenvironment is necessary for the downstream function of the PD-1 pathway, we investigated the effect of HDACi on tumor cells in the presence of immune cells. MDA-MB231 cells were exposed to vorinostat (1.5μM) up to 72 hours either alone or co-cultured with human PBMCs from healthy donors. Tumor cells and PBMCs co-cultured or cultured individually were evaluated by flow cytometry with a multiparameter panel to assess CD45, CD3, CD4, CD8 and Foxp3 expression (Supplementary Figure 6). Additionally, we quantified markers associated with immunotherapy response and prognosis in melanoma patients [27, 28], such as PD-1, PD-L1, CTLA-4 and major histocompatibility complex (MHC) class-II HLA-DR. Following vorinostat treatment, PD-L1 expression was increased in MDA-MB231 cells, which was unaffected by co-culturing with PBMCs (Figure 3B and 3C). Furthermore, these triple-negative cells exhibited a dose-dependent induction of HLA-DR after 48 hours of vorinostat treatment (Figure 3D), which was unaffected by co-culture with PBMCs (Figure 3E and 3F).

MCF-7 cells treated with vorinostat exhibited a modest increase of PD-L1, which was negligibly effected by co-culturing with PBMCs (Supplementary Figure 4B and 4C). In contrast to MDA-MB231 cells, vorinostat treatment reduced HLA-DR expression in MCF7 cells over time, which was further reduced when co-cultured with PBMCs (Supplementary Figure 4D and 4E).

### Vorinostat activity on T cells co-cultured with tumor cells

In addition to affecting cell surface expression of immune modulators on tumor cells, HDACs play a significant role in regulating immune-related genes. Foxp3 is critical for Treg development and its expression is modulated by epigenetic modifications [7]. Vorinostat treatment significantly reduces Foxp3^+^ CTLA-4^high^ Treg in PBMCs cultured alone and together with MDA-MB231 cells (Figure 3G and 3H). When co-cultured with MDA-MB231 cells, the percentage of CD4^+^ Foxp3^+^ CTLA-4^high^ T cells in PBMCs was higher than in PBMCs cultured alone. Addition of vorinostat to co-cultured MDA-MB231 cells and PBMCs attenuated this increase (Figure 3G and 3H) and reduced the total number of Foxp3^+^ CTLA-4^high^ expressing cells (Figure 3I). A reduced percentage of CD4^+^ Foxp3^+^ CTLA-4^high^ T cells in PBMCs was also observed when PBMCs were co-cultured with MCF-7 cells (Supplementary Figure 4F).

To better characterize the effects of vorinostat on PBMCs in regard to the Treg population, we performed a second analysis using a more comprehensive flow cytometry panel that includes the Treg signature markers CD25, CD127, Foxp3 and CTLA-4. 3x10^6^ PBMCs cells, obtained from healthy donors, were cultured with or without vorinostat (1.5μM) up to 48 hours. Treg were identified in the live CD4^+^ gate by high co-expression of CD25, Foxp3 and CTLA-4 and low expression of CD127. Using this gating strategy, Treg were significantly reduced upon vorinostat treatment (Figure 3J and 3K).

### Vorinostat activity on MCF-7 tamoxifen-resistant cells

**Figure 4:**
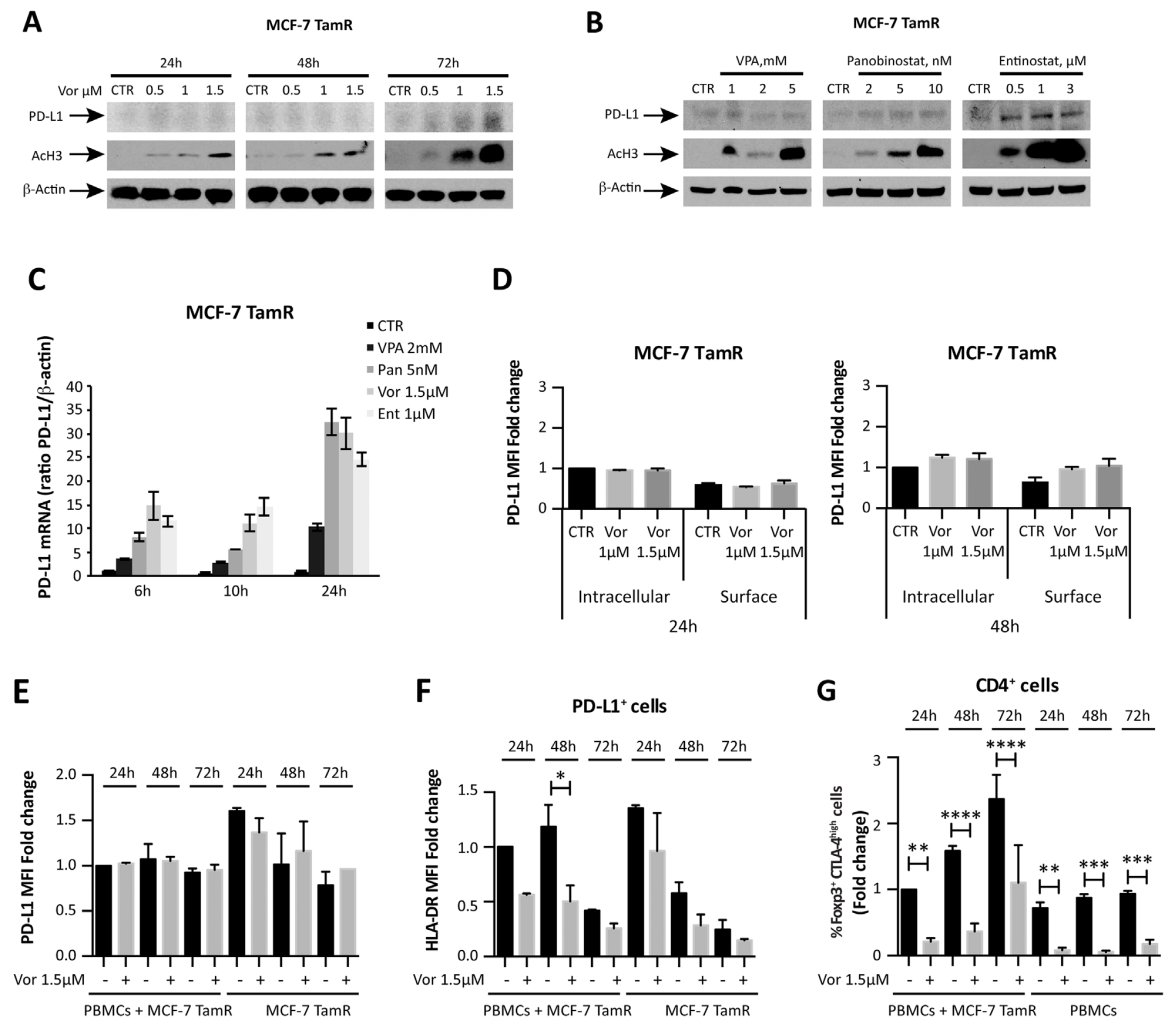
HDACi effect on tamoxifen-resistant MCF-7 breast cancer cells **(A)** MCF-7 TamR cells were untreated or treated with increasing doses of vorinostat; PD-L1 and acetyl-H3 protein expression was evaluated at 24, 48 and 72 hours after treatment by western blot analysis. **(B)** PD-L1 protein expression was evaluated by western blot in MCF-7 TamR cells exposed to VPA, panobinostat and entinostat for 48 hours. **(C)** PD-L1 mRNA expression was quantified by qReal-Time PCR in MCF-7 TamR cells after treatment with different HDACi. β-actin was used as protein loading control in western blot and housekeeping control gene to normalize qReal-Time PCR reactions. **(D)** MCF-7 TamR cells, untreated or treated with increasing doses of vorinostat for 24 and 48 hours, were collected for flow cytometry analysis, as described in the Material and Methods, to distinguish between surface and intracellular PD-L1 expression. Flow cytometric quantification of PD-L1 MFI for live tumor cells (expressed as fold change relative to the control) is shown. Flow cytometric quantification of PD-L1 **(E)** and HLA-DR **(F)** expression in MCF-7 TamR cells alone or co-cultured with PBMCs and evaluated at 24, 48 and 72 hours after treatment with vorinostat (1.5μM). **(G)** Flow cytometric quantification of CD4^+^ Foxp3^+^ CTLA-4^high^ T cells in PBMCs of healthy donors alone or in presence of MCF-7 TamR cells after 24, 48 and 72 hours of vorinostat treatment. Statistical significance is indicated by p-values as ^*^ P ≤ 0.05; ^**^ P ≤ 0.01; ^***^ P ≤ 0.001; ^****^ P ≤ 0.0001. Data are presented as the mean ± SD for (C) and ± SEM for (D-G).

Consistent with previous reports [19], we found that ER^+^ breast cancer cells have limited PD-L1 expression (Supplementary Figure 1). To further explore the relationship between HDACs function and PD-L1 expression in ER^+^ breast cancer, we investigated PD-L1 expression in a tamoxifen-resistant ER^+^ breast cancer cell line originated from MCF-7 (MCF-7 TamR) [29]. MCF-7 TamR cells expressed a higher baseline level of PD-L1 mRNA compared to its parental cells (Supplementary Figure 1B and 1D). Exposure of the resistant cells to vorinostat resulted in a modest increased PD-L1 protein expression (Figure 4A and 4B), associated with an induction of PD-L1 expression at a transcriptional level (Figure 4C). Differently from ER^-^ MDA-MB231 cells, a modest, almost undetectable, increase of PD-L1 expression was observed on the cell surface of MCF-7 TamR cells, upon vorinostat treatment for 48 hours (Figure 4D). In addition, a similar modest increase in PD-L1 MFI was observed when MCF-7 TamR cells were co-cultured with PBMCs from healthy donors (Figure 4E). In contrast to MDA-MB231, vorinostat treatment significantly reduced HLA-DR expression on MCF-7 TamR cells in the co-culture system, as observed for the parental MCF-7 cells (Figure 4F and Supplementary Figure 4E). This suggests a distinct and opposing effect of HDACi in ER^+^ and ER^-^ cells. Instead, even in the presence of MCF-7 TamR, as for the parental cells, we observed a reduction in Foxp3^+^ CTLA-4^high^ Treg upon vorinostat treatment (Figure 4G and Supplementary Figure 4F).

### HDACi-mediated increase of PD-L1 in mouse breast cancer cells

To test *in vivo* our hypothesis of HDACi as immune-priming agents, we tested PD-L1 expression in different mouse breast cancer cell lines following treatment with various HDACi. HDACi modulates PD-L1 expression in the mouse metastatic 4T1 and EMT6 breast cancer cells. In contrast, PD-L1 down-regulation or no effect was seen in JC mouse breast cancer cells following HDACi treatments (Supplementary Figure 7A). Consistent with human breast cancer cells (Figure 2B), PD-L1 up-regulation was increased when vorinostat was combined with azacitidine in the 4T1 and EMT6 mouse cell lines (Supplementary Figure 7B).

### *In vivo* antitumor effect of vorinostat in combination with immunotherapy

To evaluate the potential interaction of HDACi to prime immunotherapy *in vivo*, the highly proliferative and resistant 4T1 mouse breast cancer cell line was chosen, the only known mouse cell line with similar immunogenicity, metastatic properties and growth characteristics as TNBC [30]. To induce tumor formation, 4T1 cells were subcutaneously implanted in BALB/c mice. Once tumors formed, mice were randomly assigned to receive doses of vorinostat, anti-PD-1 (a-PD-1) blockade, both drugs, or the vehicle as a control. A significant benefit was not observed when vorinostat was combined to the anti-PD-1 blockade in regard to tumor growth or survival compared to single-drug treatment (Supplementary Figure 8A and 8B). This is consistent with previous reports showing that the 4T1 tumor model is highly resistant to most therapeutic strategies, including checkpoint inhibitor blockade [30-32].

**Figure 5:**
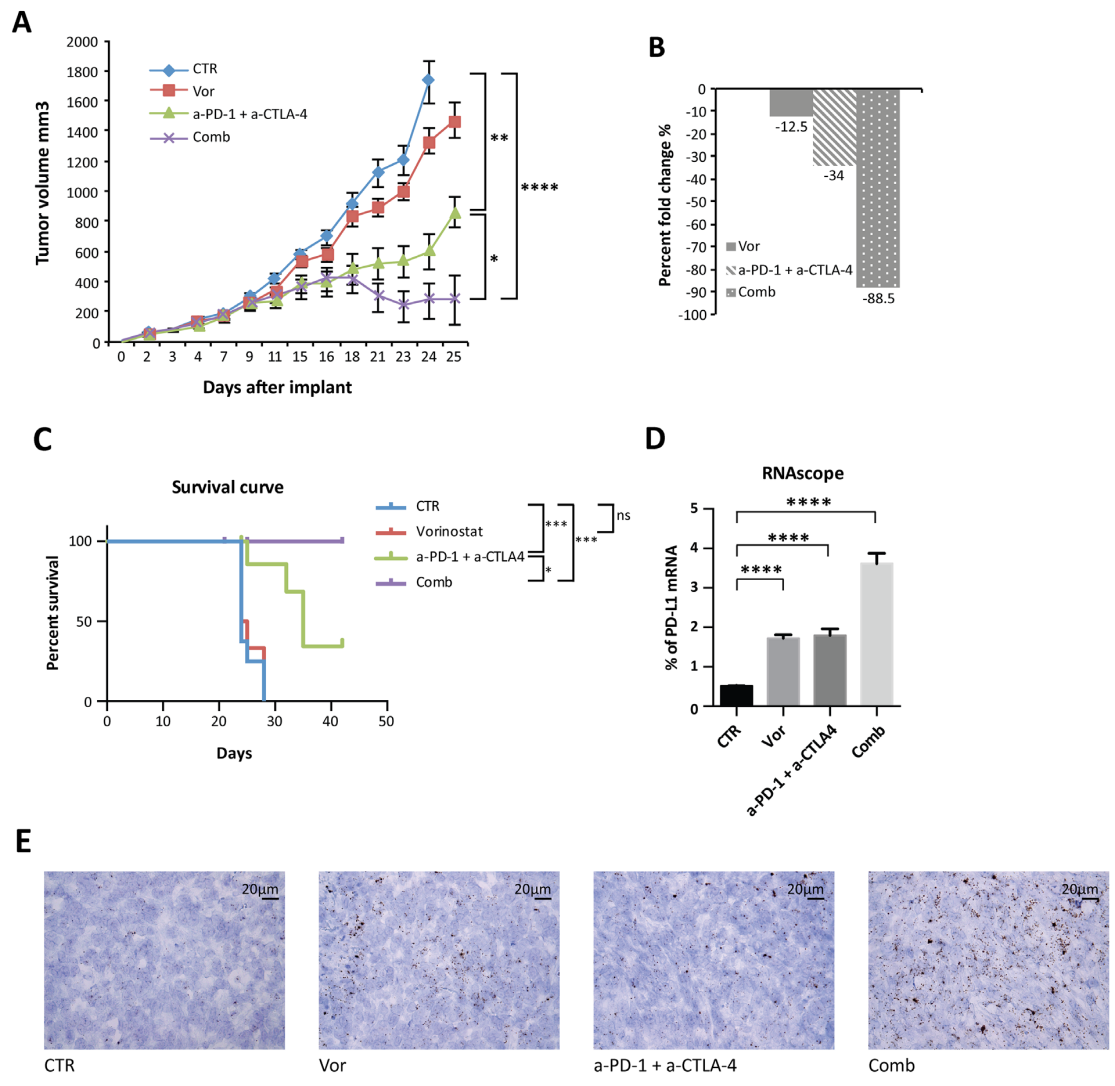
Anti-tumor activity of vorinostat, anti-PD-1 and anti-CTLA-4 on established mouse breast cancer allografts **(A)** 4T1 cells (1 x 10^6^) were s.c. injected into BALB/C mice. When established tumors were palpable, mice were treated with vorinostat (100 mg/kg i.p.), anti-PD-1 (10 mg/Kg i.p.) combined with anti-CTLA-4 (10mg/Kg i.p.) or a combination of the three drugs as described in the Material and Methods. Relative tumor volume curves for 4T1 allograft; measurements are shown as mean ± SEM tumor volume (n = 8). **(B)** Tumor volume averages from each group at day 0 and day 25 (end of treatment) were compared and presented as percentages of vehicle. **(C)** Effect of vorinostat and/or anti-PD1 + anti-CTLA-4 treatments on the survival of 4T1 allograft mice. **(D)** Mouse PD-L1 mRNA was measured in FFPE 4T1 tumor samples by RNAscope assay. Hybridization signals were amplified and visualized with RNAscope 2.0 HD detection kit. Data are presented as the mean ± SEM. **(E)** Murine PD-L1 RNAscope images were captured under a bright field at 40x magnification. One representative image for each treatment is shown. Positive signals showed as brown punctuate dots were analyzed by scoring with ImageJ software. Statistical significance is indicated by p-values as ^*^ P ≤ 0.05; ^**^ P ≤ 0.01; ^***^ P ≤ 0.001; ^****^ P ≤ 0.0001; Ns: non significant.

In order to overcome this resistance and to achieve a wider immune-modulatory effect, anti-CTLA-4 (a-CTLA-4) blockade was added to the therapeutic regimen to better represent what has been described in clinic. Indeed, even if both PD-1 and CTLA-4 function as T cells negative regulator, emerging data point to their non-redundant role in modulating immune response [12, 33]. Mice were injected with vorinostat, a combination of anti-PD-1 and anti-CTLA-4 blockades, the three drugs in combination, or the vehicle as a control and tumor volume and percent change were measured (Figure 5A and 5B). The addition of anti-CTLA-4 to the anti-PD-1 blockade induced a significant inhibition of tumor growth compared to single-agent treatment, which was significantly improved by vorinostat addition (Supplementary Figure 8A and Figure 5A). Vorinostat, the immunotherapy combination (a-PD-1 + a-CTLA-4) and the three-drugs combination reduced the tumor burden by 12.5%, 34% and 88.5%, respectively (Figure 5B). Although repeated treatment with anti-CTLA-4 and anti-PD-1 as single-agent retarded tumor growth, tumor eradication was only observed when the HDACi was combined with the immunotherapy treatment. This synergistic interaction of the three drugs resulted in a significant survival increase (Figure 5C). Notably, the combined treatment was well tolerated (Supplementary Figure 8C).

### Pharmacodynamic effects in allograft tumors

Following treatments, tumors were harvested and evaluated. PD-L1 tumor expression was evaluated by RNA *in situ* hybridization analysis on formalin fixed paraffin embedded (FFPE) tissues. Indeed PD-L1 immunohistochemistry (IHC) implicates several divergences in results interpretation, due to the absence of standardization and universal definition of positive cut-off, specificity and reproducibility of the available antibodies. Consistent with *in vitro* data, a significant PD-L1 mRNA up-regulation was observed with vorinostat treatment, further increased in tumors treated with the three-drug combination (Figure 5D and 5E).

**Figure 6:**
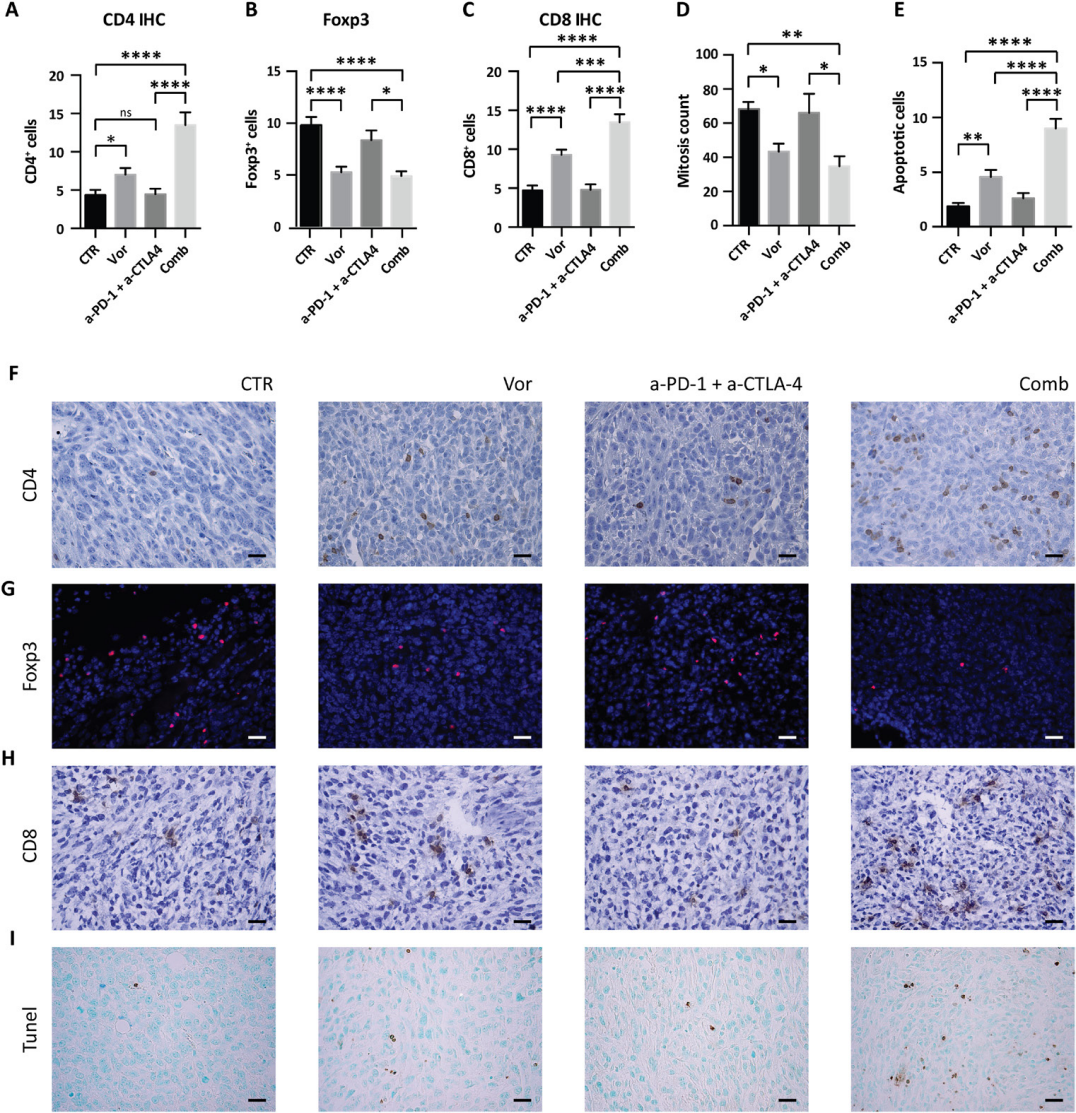
Effect of vorinostat/immunotherapy treatment on immune cell subset, proliferation and apoptosis in 4T1 allograft tumors Paraffin-embedded tissues were generated from each tumor for IHC and immunofluorescence analysis. Slices were stained for CD4 **(A)**, Foxp3 **(B)** and CD8 **(C)** by IHC or immunofluorescence. Mitotic counts **(D)** were performed on H&E-stained sections, while apoptosis was measured by TUNEL assay **(E)**. Data are presented as the mean ± SEM. Statistical significance is indicated by p-values as ^*^ P ≤ 0.05; ^**^ P ≤ 0.01; ^***^ P ≤ 0.001; ^****^ P ≤ 0.0001; Ns: non significant. Representative image for CD4 **(F)**, Foxp3 **(G)**, CD8 **(H)**, and TUNEL assay **(I)** are shown. Images were captured with a 40x objective on a light microscope. Scale bars correspond to 20μm. Scale bars correspond to 20μm.

Insufficient TILs and CD8^+^ T cells tumor infiltrate is recognized as one of the mechanism involved in immunotherapy resistance. We hypothesized that increasing T cells infiltration into tumors could be an efficacious strategy to enhance immunotherapy response. Thus, the number of CD4^+^ and CD8^+^ T cells was quantified in the tumors by IHC. Tumors treated with vorinostat had significantly more CD4^+^ T cells compared to the vehicle group, which was further increased in the presence of immunotherapy (Figure 6A and 6F).

CD4^+^ T cells commonly include Treg and conventional T helper (Th) cells. While Th cells control adaptive-immunity releasing cytokines that activate other effector immune cells against pathogens and cancer, Treg are suppressor T cells that modulate the immune system by suppressing induction and proliferation of effector T cells [34]. As Treg presence in tumors is associated with a poor prognosis [35], Foxp3 expression was evaluated in tumors to differentiate the increase in CD4^+^ T cells from an increase in Treg. Immunofluorescence staining of Foxp3 showed that vorinostat decreased the number of Foxp3^+^ cells, whereas the addition of immunotherapy had no effect (Figure 6B and 6G). In contrast, we found an increase in CD8^+^ T cells after vorinostat treatment that was further induced in tumors receiving the triple-combination treatment, consistent with an induction of cytotoxic T lymphocytes in the tumor microenvironment (Figure 6C and 6H).

Tumor proliferation and survival were evaluated to determine the impact of HDACi on tumor growth. Vorinostat treatment induced a significant reduction in the number of mitotic cells alone and in combination with immunotherapy (Figure 6D). This was associated with a decrease in Ki67 expression. Indeed a trend in the reduction of Ki67 was found when vorinostat was administrated alone or in combination (Supplementary Figure 9A). A significant increase of apoptosis was observed by TUNEL assay in tumors receiving vorinostat compared to those receiving vehicle, which was further intensified when combined with immunotherapy (Figure 6E and 6I). The percentage of necrotic cells was not significantly different in tumors receiving vorinostat compared to the combined treatment (Supplementary Figure 9B).

Taken together these data confirmed the *in vitro* findings that PD-L1 modulation by HDACi is associated with increased TILs in the tumour microenvironment, which could explain the increased efficacy observed in the combination treatment *in vivo*.

## DISCUSSION

Immune checkpoint inhibitors activate exhausted T cells in several type of cancer, including TNBC [36]. Although they have been successfully integrated in the treatment of cancer, their role and optimal placement in breast cancer remains uncertain. Several reports have linked higher PD-L1 expression in breast cancer to better response and survival [16-19].

Although the presence of PD-L1 may enrich for response, in breast cancer this may not be sufficient. Previous reports have shown that HDACi modulate immune response, alter Treg activity and regulate cytokine expression [6, 7, 37, 38]. In this study, we report a rational and innovative therapeutic approach in breast cancer that combines immunotherapy with HDACi, which acts in part by priming anti-tumor immune response. Our data suggest that HDACi mediated up-regulation of PD-L1 and HLA-DR in TNBC may promote tumor recognition through the T cell receptor, thus enhancing the immune response.

Tumors evade immune surveillance by acquiring immunosuppressive phenotypes, modifying immune checkpoint pathways, recruiting Treg and avoiding cytotoxic T cells and antigen presenting cells recognition of the tumor cells. MHC class-I and –II are responsible for these interactions and their down-regulation is associated with immune suppression, poor prognosis and metastatic progression. In particular, HLA-DR has an important role in Th/inducer lymphocytes proliferation and low HLA-DR expression in breast cancer is associated with a mechanism of escape from Th-mediated immune response and increased tumor metastasis [39]. HLA-DR expression was found to be a promising positive predictive factor in node-negative breast cancer [40]. Similar results were found in colorectal cancer and in melanoma, where HLA-DR expression was associated with therapeutic response to immunotherapy, better progression free survival, overall survival (OS) and CD4^+^ and CD8^+^ T cells tumor infiltrate [28, 41]. MHC-II^+^ epithelial cells can present antigen to CD4^+^ Th cells and thus, promoting the expression of HLA-DR in tumor cells with HDACi, may promote anti-tumor immunity and tumor suppression as an adaptive response.

Our *in vivo* data show that vorinostat combined with immune checkpoint inhibitors decreased overall tumor growth, prolonged survival and, in some cases, completely eradicated the tumor. We postulate that the therapeutic benefit observed in our study is a consequence of vorinostat-induced increase in tumor cells immunogenicity through HLA-DR up-regulation, associated with increase in T cells recognition, function and activation by anti-PD1 and anti-CTLA-4 blockades. Our *in vivo* studies further show the relative ineffectiveness of PD-1 blockade alone and the necessity to co-inhibit both the PD-1 and CTLA-4 pathway to have a significant anti-tumor effect. This is consistent with a recent study where the success of anti-PD1 strategy requires the CD28/B7 pathway co-stimulatory function [42]. Blocking CTLA-4 may be necessary to allow CD28-dependent CD8^+^ T cells activation and proliferation after PD-1 blockade [42]. Furthermore, HDAC inhibition decreases Foxp3 expression in CD4^+^ T cells, which may increase an anti-tumor immune response by down-regulating suppressive Treg activity. Several reports have linked the increased Treg presence in breast tumors with an invasive phenotype and diminished OS [43-45]. Significant reduction in primary and metastatic tumor progression was obtained with Treg ablation in an oncogene-driven mammary carcinoma [46]. Moreover, increased frequency and higher proliferative activity of Treg has been correlated with higher tumor grade breast cancer [47]. These reports corroborate the importance of our finding and, together with HDACi-dependent promotion of T cells infiltration in the tumor microenvironment, may explain the increased efficacy of this combinational strategy.

CCL5 is a potent chemotactic for T cells, monocytes and eosinophils and plays an active role in recruiting leukocytes into inflammatory sites [48]. Highly expressed in various tumors, CCL5 promotes tumor growth and metastasis by inducing tumor cell proliferation, angiogenesis and matrix metallo-proteinases in breast cancer [48, 49]. CCL5 was found to reduce anti-tumor immune response by increasing the presence of tumor-associated macrophages and Treg [49, 50]. A previous report demonstrated that the HDACi romidepsin enhances response to PD-1 blockade in lung cancer by inducing the expression of CCL5, CXCL9 and CXCL10, and thus enhancing T cells infiltration [20]. Conversely, another report in colorectal cancer associated a higher expression of CCL5 with a greater number of Treg and increased CD8^+^ T cells apoptosis, suggesting a critical role for CCL5 in recruiting Treg and enhancing their ability to suppress CD8^+^ T cells [49]. We found that MDA-MB231 cells were characterized by a very low, almost undetectable, expression of CXCL9 and CXCL10 mRNA, only modestly affected by HDAC inhibition (data not shown), whereas the HDACi induced a significant down-regulation of CCL5 (Supplementary Figure 10). We believe that this may be linked to the reduction of CD4^+^ Foxp3^+^ cell infiltration observed in our study, as also postulated by others [49].

Opposite to the effects seen in TNBC cells, PD-L1 basal expression was low in ER^+^ breast cancer and was only modestly up-regulated with treatment. This effect did not translate in PD-L1 up-regulation on ER^+^ cells surface (Figure 4D and Supplementary Figure 4A). Furthermore, HLA-DR was down-regulated, which would further support the non-immunogenic behavior of ER^+^ cells. Hormone responsive tumors are, indeed, known to be less immune response driven [36]. PD-L1 baseline expression was more pronounced in the ER^+^ tamoxifen-resistant cells, but its expression was modestly increased with HDAC inhibition, not at the cell surface level, and yet not associated with HLA-DR up-regulation. Our data suggest that HDAC inhibition increases the immunogenicity of TNBC, increasing PD-L1 and HLA-DR expression, and reduces the immune system inhibitory compartment, decreasing CD4^+^ Foxp3^+^ Treg. This assumes a particular significance in the TNBC tumor characterized by an already existing, but masked immunogenicity that can be boosted by HDACi. On the other hand in ER^+^ tumors, characterized by a non-immunogenic behavior, the attempt to increase the tumor–immune system recognition by HDACi fails to succeed due to their intrinsic unresponsiveness and the absence of pre-exiting T cells infiltration in the tumors.

**Figure 7:**
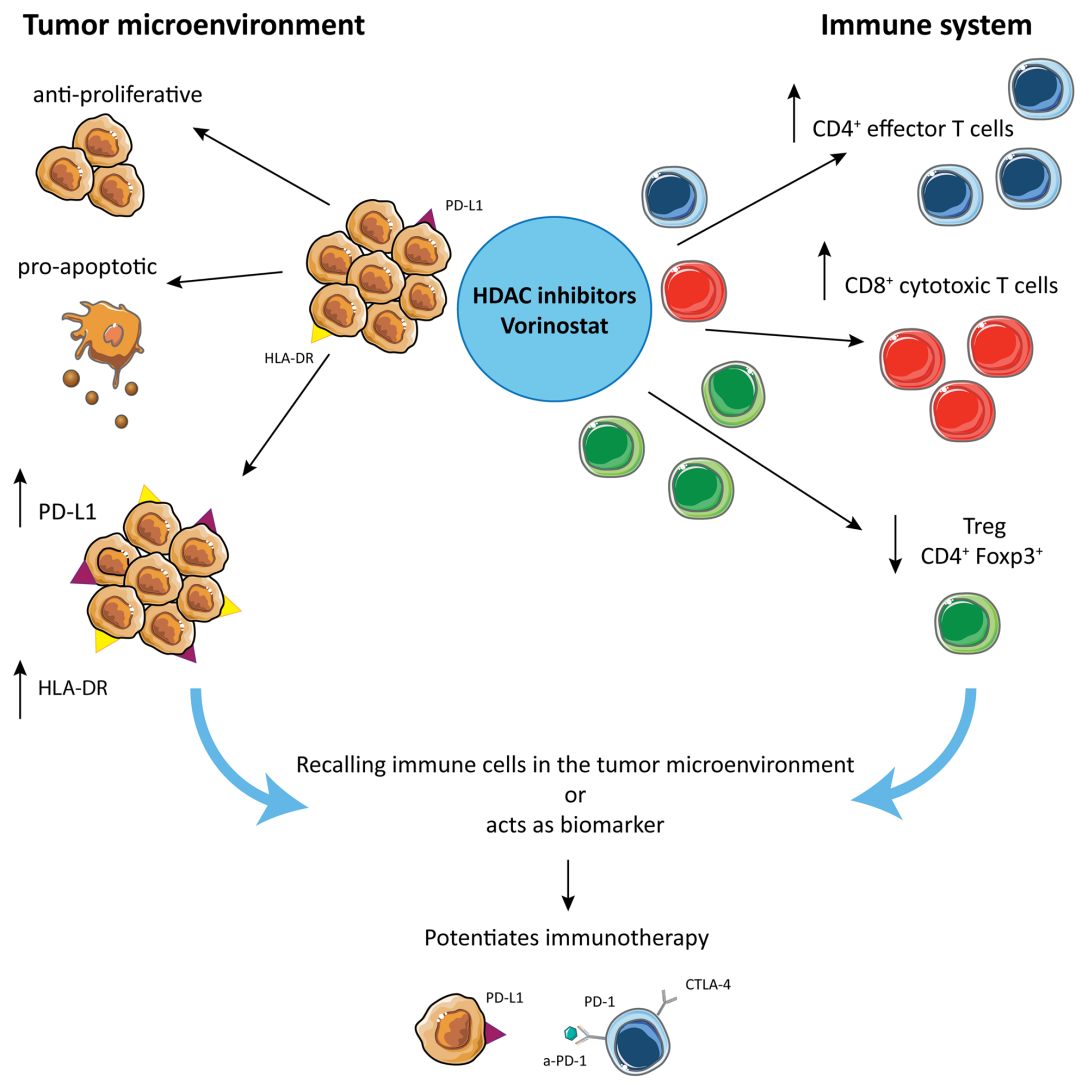
Hypothetical mechanism by which HDACi potentiate checkpoint inhibitors treatment in TNBC HDACi are responsible for multiple different effects: on one side they induce anti-proliferative and pro-apoptotic effects on tumor, while they induce PD-L1 and HLA-DR expression on tumor cells, making the tumor more susceptible for tumor-antigen recognition. On the other hand, HDACi increase CD4^+^ and CD8^+^ T cell tumor infiltration and reduce the frequency of Treg in the tumor microenvironment.

In conclusion, HDAC inhibition increases PD-L1 and HLA-DR expression in TNBC and decreases Treg frequency, which when combined with PD-1 and CTLA-4 blockade promotes TILs infiltration, tumor apoptosis, tumor regression and increased survival in mice (Figure 7). There are several possible explanations for these findings. The increased PD-L1 expression in TNBC could be either a) an isolated functional marker whose presence and up-regulation indicate increased sensitivity to the PD-1/PD-L1 pathway; which can be disrupted by the addition of checkpoint inhibitors or b) a marker of global changes in JAK/STAT induced gene expression, that in turn leads to increased sensitivity to checkpoint inhibitors seen in the TNBC model. In either scenario, the HDACi induction of PD-L1 can support the dependence of the tumor to the PD-1/PD-L1 pathway.

Overall, this study suggests that the combination of HDACi with immune checkpoint inhibitors identifies a novel therapeutic strategy for the treatment of TNBC.

## MATERIALS AND METHODS

### Reagents

VPA was purchased from Enzo Life Sciences. Stock solutions were prepared in sterile water. Entinostat (MS-275) and vorinostat were from Selleck Chemicals; panobinostat (LBH589) from Novartis International; 5-Azacytidine (azacitidine) was from Sigma; GSK126 was from Active Biochem. Stock solutions were prepared in DMSO. Monoclonal antibodies anti-mouse PD-1 (clone RMP1-14, #BE0146) and anti-mouse CTLA-4 (clone 9H10, #BE0131) were purchased from Bioxcell. Actinomicin D was purchased from Sigma Aldrich.

All media, serum, antibiotics, and glutamine were from Corning.

Primary antibodies (Abs) for western blotting: β-Actin-Ab (Sigma-Aldrich, cod.A5316), Programmed death-ligand 1 (PD-L1)-Ab (Abcam, cod.Ab58810); (PD-L1)-Ab (cod.#13684), acetyl-H3-Ab (cod.#9649), PARP-Ab (cod.#9542) (Cell signaling Technology), and acetyl-H4-Ab (Millipore cod.06946). For IHC: monoclonal anti-mouse Ki67-Ab (Cell signaling Technology; cod.#12202), monoclonal anti-mouse CD4-Ab (Abcam, cod.Ab183685), anti-mouse CD8-Ab (eBioscience, clone 56-6.7). For immunofluorescence on fresh frozen tissues: anti-Foxp3-efluor570 (eBioscience clone FJK-16s, #41-5773-80).

### Cell culture conditions

The MCF-7, MCF-7 TamR, T47D and SKBR3 cells were maintained in DMEM, whereas 4T1, JC mouse breast cancer cells and MDA-MB231 in RPMI-1640 medium. EMT6 were maintained in Waymouth MB 752/1 media with 85% glutamine 2mM, 15% FBS. All cell lines were cultivated as described before [23]. MCF-7, T47D, SKBR3, MDA-MB231, JC and EMT6 were obtained from American Type Culture Collection, while 4T1 were kindly provided by Dr B. Hann. MDA-MB231, MCF-7 and T47D cells have been authenticated with a short tandem repeat profile on Promega PowerPlex16HS Assay at University of Arizona Genetics Core.

### Western blot analysis

Cells were lysed in RIPA buffer (Sigma) and separated by SDS poly-acrylamide gel electrophoresis. Proteins were transferred to PVDF membranes and immunoblotted with the previous indicated antibodies. Immunoreactive bands were detected by enhanced chemiluminescence (GE Healthcare, cod. RPN2232), after the blots were probed with the appropriate horseradish peroxidase-linked IgG antibodies.

The signal intensities of a protein band and its surrounding background were scanned from images derived from at least two independent Western blot experiments for each cell line and quantified by using ImageJ software. Western blot relative quantifications of PD-L1 protein expression was normalized to β-actin and plotted as fold change.

### Real-time quantitative PCR (qReal-Time PCR)

Total RNA was extracted from the cells by Trizol reagent according to the manufacturer’s instructions (Invitrogen). The reverse transcription-PCR (RT-PCR) assay was performed with iScript cDNA Synthesis Kit (Biorad).

Human PD-L1 (Hs01125301_m1), human GADD45a (Hs00169255_m1) and mouse PD-L1 (Mm00452054_m1) mRNA expression were quantified by the 5′-nuclease method using StepOnePlus™ Real-Time PCR Systems (Applied Biosystems). Each gene was tested in each cell line in triplicates in three independent experiments. The relative changes in gene expression were normalized to endogenous human or mouse β-actin gene expression levels respectively (Human ACTB and mouse Mm00607939_s1, Applied Biosystems) by the −2^ΔΔCT^ method.

To investigate the mechanism by which vorinostat regulates PD-L1 mRNA, MDA-MB231 cells were untreated or treated with 5 μg/ml actinomycin D and/or vorinostat (1.5μM) up to 10 hours. PD-L1 and GADD45a mRNA expression was determined as described above. When analyzing the combination of vorinostat with other epigenetics drugs, MDA-MB231 cells were cultured in the presence of vorinostat 1.5μM in combination with azacitidine 2μM and in combination with GSK126 300nM for 24 hours.

### Immunofluorescent staining for PD-L1

MDA-MB231 or MCF-7 cells were seeded 30000 cells/well. PD-L1 protein expression was evaluated after 48 hours of treatment with vorinostat (1.5μM). Cells were stained for PD-L1 antibody (red, Cell signaling Technology cod.#86744) according to the manufacturer’s instructions. After secondary antibody incubation (Alexa Fluor 555, Invitrogen cod.A31572), slides were mounted with a DAPI (blue) mounting media (ProLong® Gold Antifade Mountant with DAPI, Life Technologies). Slides were next analyzed by microscopy (Zeiss AxioImager M1, Zeiss). Representative images show PD-L1^+^ cells with 20x and 40x magnification.

### Co-culture experiments and flow cytometry analysis

3x10^5^ MDA-MB231 cells were seeded in the presence or absence of 3x10^6^ PBMCs cells freshly obtained by healthy donor blood (ratio 1:10). Tumor cells, PBMCs or co-cultured cells were treated or untreated with vorinostat (1.5μM) and collected after 24, 48 or 72 hours to perform multiparameter flow cytometry analysis. The following human Abs were used: anti-CD3 (#317324), anti-PD-1 (#329908), anti-CD45 (#304049), anti-CD8 (#301039) Biolegend; anti-CD4 (#46-0047-42), anti-CTLA-4 (#12-1529-42), anti-HLA-DR (#47-9956-41), anti-Foxp3 (#48-4777-42) eBioscience; anti-PD-L1 (#558017), anti-Ki67 (#561277) BD Biosciences; Ghost Violet 510 viability dye (#13-0870 Tonbo Bioscence). Gates were determined using FMO and isotype control Ab staining. Data are expressed as mean of at least three separate experiments ± the standard error of the mean (± SEM). Similar experiments in co-culture were performed for MCF-7 and MCF-7 TamR cells. A similar flow cytometry panel was used for the dose-response experiment and to investigate the surface/intracellular position of PD-L1 on MDA-MB231 and MCF-7 TamR cells.

To investigate if vorinostat was able to modify the Treg compartment, 3x10^6^ PBMCs cells, obtained from healthy donor blood, were treated or not with vorinostat (1.5μM) and collected after 24 and 48 hours to perform multiparameter flow cytometry analysis. The following human Abs were used: anti-CD3 (#56-0038-42), anti-CD25 (#46-0257-42), anti-CD127 (#47-1278-42), anti-CTLA-4 (#12-1529-42), anti-Foxp3 (#48-4776-42) eBioscience; anti-CD4 (#317438) and anti-CD8 (#301042); Ghost Violet 510 viability dye (#13-0870 Tonbo Bioscence). Data are expressed as mean of two separate experiments repeated on four healthy donors ± SEM.

Data were acquired by an LSRFortessa (BD Biosciences) and analyzed using FlowJo software (Tree Star Inc.). To standardize voltages over time, Sphero Ultra Rainbow Beads (Spherotech) were used to calibrate and normalize to baseline intensity.

### *In vivo* allograft studies

Four- to six-week-old female BALB/C mice (Harlan Laboratories) were acclimatized in the Laboratory Animal Resource Center at UCSF in accordance with the institutional guidelines (protocol AN136635). 4T1 cells (1x10^6^) diluted in RPMI (without P/S and FBS) were implanted subcutaneously (*s.c*.) in the flank region of the mice. When the tumors became palpable, thirty-two mice were randomized into four experimental groups (*n*=8). Based on previous studies (Supplementary Figure 5 and [20, 23, 51]), the mice were treated intra peritoneal (*i.p.*) with 10 mg/kg anti–PD-1 and anti–CTLA-4 antibodies (diluted in sterile PBS) on days 4, 6, 8, 10 post-tumor implantation and with vorinostat (100 mg/kg melted in DMSO and diluted daily in 10%DMSO+45%PEG-400+45%PBS) *i.p.* on days 5, 7, 9, 11. This schedule was repeated for a total of 22 days. Control group mice were treated with vehicle (PBS/PEG-400) solution.

Tumor volume (mm^3^) was calculated as described before [23]. The mice were weighted three times/week while monitored daily for clinical signs and mortality. Mice were sacrificed when tumors reached a volume ≥ 1cm^3^, a length ≥ 2cm or got ulcerated. At the end of the study, the remaining mice were sacrificed by exposure to CO_2_ followed by cervical dislocation. The percent change in the experimental groups was compared with that of the vehicle control group, as previously described [23].

### RNA *in situ* hybridization of PD-L1

Mouse PD-L1 mRNAs were measured in 5μm FFPE 4T1 tumor samples by RNAscope assay (Advanced Cell Diagnostics, ACD) following the manufacturer’s instructions. Briefly, sections were deparaffinized, incubated with pretreatment reagents and target-retrieval was performed. FFPE samples were hybridized with m-CD274-probe (ACD) and hybridization signals were amplified with RNAscope 2.0 HD detection kit (Brown, ACD). Images were captured under a bright field at 40x magnification. Positive signals showed as brown punctate dots, were analyzed by scoring with ImageJ software. To check tissue RNA stability and non-specific hybridization, m-Ppib and DapB were used as positive and negative probes (ACD).

### Histology, IHC, immunofluorescence analysis from mice tumor samples

At the end of treatment, at least two mice per group were sacrificed for pharmacodynamic studies. For immunohistochemical analysis, mitotic counts were performed on hematoxylin and eosin (H&E)-stained sections and calculated as an average of two representative high power fields. Expression of Ki67, CD8 and CD4 was determined by IHC. Briefly, sections were incubated with primary antibody and then with SignalStain Boost IHC Detection Reagent (Cell signaling Technology) as secondary antibody for Ki67 and CD4, and with HRP Rat-on-mouse secondary antibody kit (Biocare) for CD8. Peroxidase reactivity was visualized using a 3,3’-diaminobenzidine (Vector Laboratories, cod.SK4100). A single pathologist (G.K.) performed a blinded analysis of the slides for Ki67 and for the mitotic count.

Apoptosis was evaluated by TUNEL assay according to the manufacturer’s protocol (FragEL™ DNA Kit #QIA33, Calbiochem).

Foxp3 immunofluorescence staining was performed on fresh frozen tissues. Briefly 10μm thick tissue slides were fixed with a 1:1 solution of methanol/acetone, blocked with 3% BSA+1:200 mouse BD FC block (clone 2.4G2, BD) in PBS. Slides were exposed to anti-Foxp3-efluor570 antibody (red) and mounted with DAPI (blue). Representative images show Foxp3^+^ cells with 40x magnification.

### Statistical analysis

Representative results from a single experiment of qReal-TimePCR (standard deviation of triplicates is shown in the figures), western blot, immunofluorescence, *in situ* hybridization and IHC are presented; additional experiments yielded similar results. Data are expressed as mean with standard deviation (± SD) or ± SEM indicated.

Appropriate statistical analyses were applied, assuming a normal sample distribution. Statistical significance in the differences of tumor growth *in vivo* was determined by the One-way Anova Test, followed by Bonferroni post-test (^*^ P ≤ 0.05; ^**^ P ≤ 0.01; ^***^ P ≤ 0.001; ^****^ P ≤ 0.0001). Kaplan-Meier survival curves were analyzed with a log-rank test (^*^ P ≤ 0.05; ^***^ P ≤ 0.001; Ns: non significant). Mitotic count differences, RNAscope, CD4, CD8 IHC, Foxp3 immunofluorescence, flow cytometry experiments and TUNEL assay results were analyzed by One-way Anova followed by Bonferroni post-test (^*^ P ≤ 0.05; ^**^ P ≤ 0.01; ^***^ P ≤ 0.001; ^****^ P ≤ 0.0001; Ns: non significant). All statistical evaluations were performed with Prism software (GraphPad Software, Inc).

### Ethics approval

Animal studies were conducted according to a UCSF Laboratory Animal Resource Center (LARC) protocol (AN136635). This protocol was approved by the UCSF Institutional Animal Care and Use Committee (IACUC) accredited by Association for Assessment and Accreditation of Laboratory Animal Care International (#001084).

## SUPPLEMENTARY MATERIALS FIGURES



## Abbreviations

CCL5: C-C motif chemokine ligand 5
CTLA-4: cytotoxic T-lymphocyte-associated protein 4
CXCL9: C-X-C motif chemokine ligand 9
CXCL10: C-X-C motif chemokine ligand 10
DNMTi: DNA methyltransferase inhibitor
ER: estrogen receptor
EZH2: Enhancer of zeste homolog 2
FFPE: formalin fixed paraffin embedded
FMO: fluorescence minus one
Foxp3: forkhead box P3
HDAC: histone deacetylase
HDACi: histone deacetylase inhibitor
Her2: human epidermal growth factor receptor 2
HLA-DR: Human Leukocyte Antigen - antigen D Related
IHC: immunohistochemistry
MCF-7 TamR: MCF-7 tamoxifen-resistant cells
MFI: mean fluorescent intensity
MHC: major histocompatibility complex
OS: overall survival
PBMCs: peripheral blood mononuclear cells
PD-1: Programmed cell death protein 1
PD-L1: Programmed death-ligand 1
PR: progesterone receptor
SD: standard deviation
SEM: standard error of the mean
TILs: tumor-infiltrating lymphocytes
TNBC: triple negative breast cancer
Treg: regulatory T cells
