# Supplementary material for: HDAC inhibition potentiates immunotherapy in triple negative breast cancer
Source: Oncotarget. 2017 Dec 12;8(69):114156–72. doi: 10.18632/oncotarget.23169 (PMC5768393; doi:10.18632/oncotarget.23169)
Supplement: Supplementary file 1 [file oncotarget-08-114156-s001.pdf]

HDAC inhibition potentiates immunotherapy in triple negative breast cancer

SUPPLEMENTARY MATERIALS

Gene-knockout

RNA interference (siRNA) against human HDAC1, HDAC3, HDAC6 (Integrated DNA Technologies), or HDAC2 (Ambion, Life Technologies) were transiently transfected for 24 hours into MDA-MB231 cells using Lipofectamine 2000 (Invitrogen) according to the manufacturer's instructions. Scrambled negative control DsiRNA (Integrated DNA Technologies) and Silencer Select Negative Control 1 (Ambion, Life Technologies) were used as the negative control respectively. Human HDAC1 (Hs02621185_s1), HDAC2 (Hs00231032_m1), HDAC3 (Hs00187320_m1), HDAC6 (Hs00195869_m1) mRNA expression was quantified as previously described.

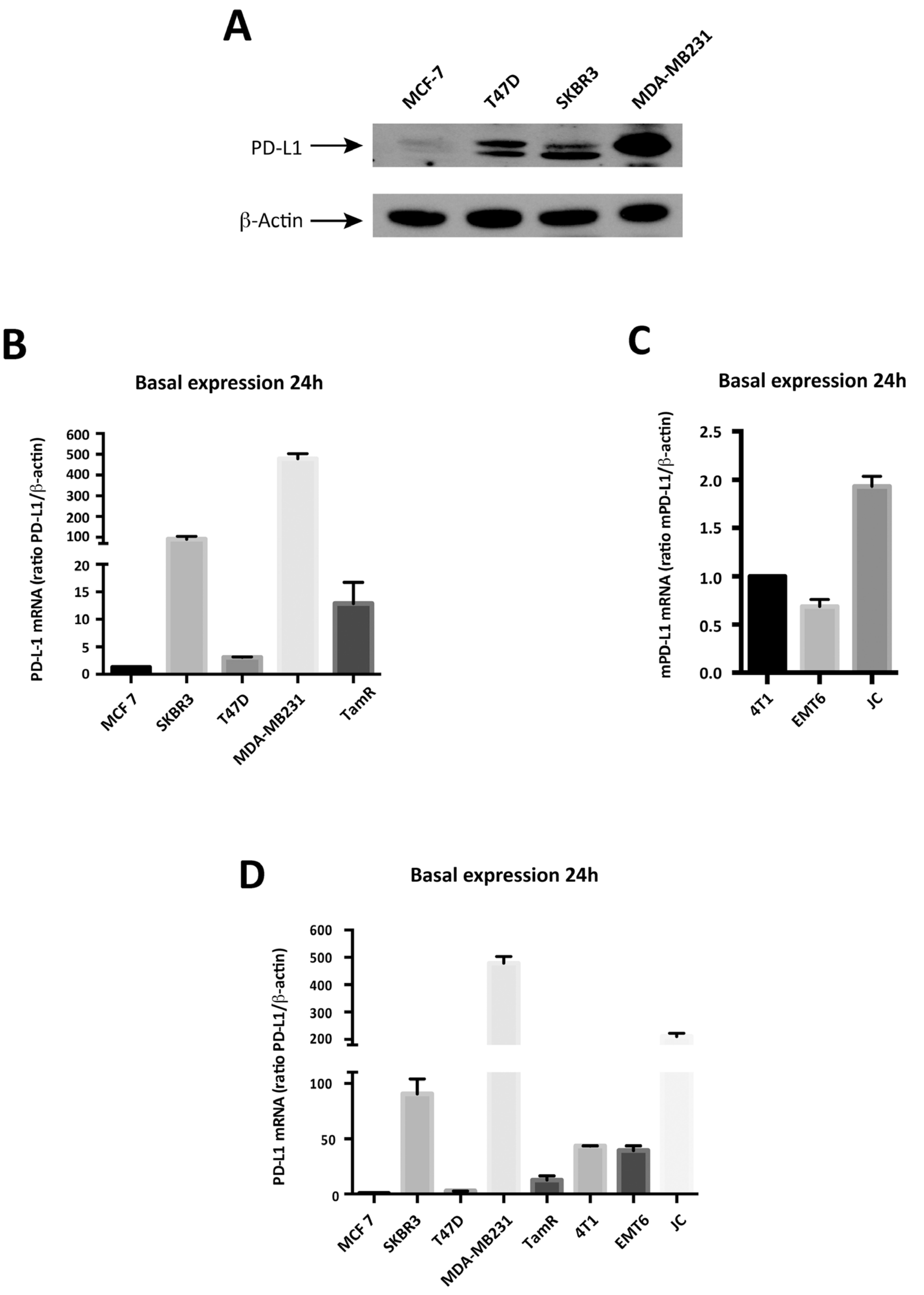

Supplementary Figure 1: TNBC cells are characterized by a higher basal PD-L1 expression compared to other breast cancer cell lines. **(A)** Basal expression of PD-L1 protein evaluated by western blot in MCF-7, SKBR3, T47D and MDA-MB231 cells. β-actin was used as protein loading control. **(B)** Human PD-L1 basal mRNA expression was determined by qReal-Time PCR in MCF-7, SKBR3, T47D, MDA-MB231 and MCF-7 tamoxifen resistant (MCF-7 TamR) cells. **(C)** PD-L1 basal mRNA expression was evaluated in mouse breast cancer cell line 4T1, EMT6 and JC. **(D)** PD-L1 mRNA expression compared between human and mouse cell lines. β-actin was used as housekeeping control gene to normalize qReal-Time PCR reactions. Data are presented as the mean ± SD.

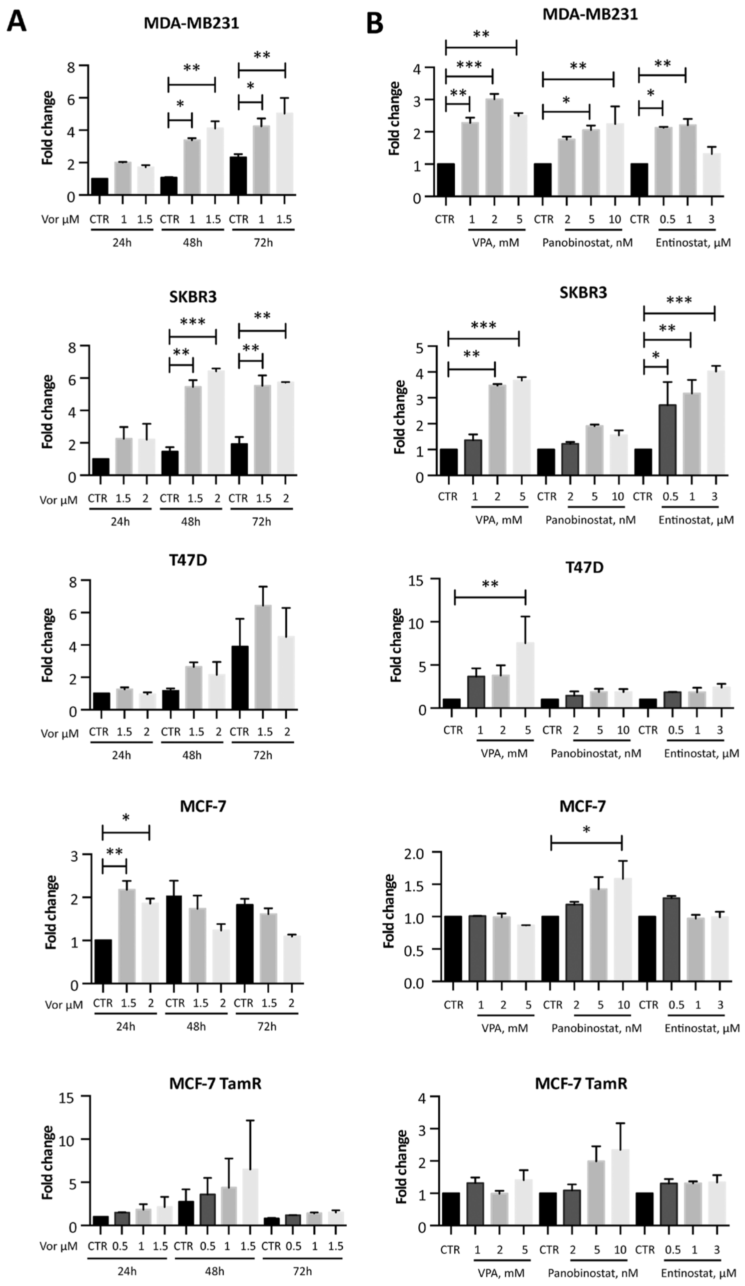

Supplementary Figure 2: Western blot relative quantifications of PD-L1 protein expression normalized to β-actin from MDA-MB231, SKBR3, T47D, MCF-7 and MCF-7 TamR cells before and after HDACi treatment. (A) Breast cancer cells were treated or not for 24, 48 and 72 hours with increasing doses of vorinostat. **(B)** Breast cancer cells were untreated or treated for 48 hours with valproic acid (VPA), panobinostat and entinostat. The signal intensities of a protein band and its surrounding background were scanned from images derived from at least two independent Western blot experiments for each cell line and quantified by using ImageJ software. The resultant values of protein expression were normalized to those of β-actin and then plotted as fold change relative to the control at 24 hours for (A) and to the relative control at each time point in (B).

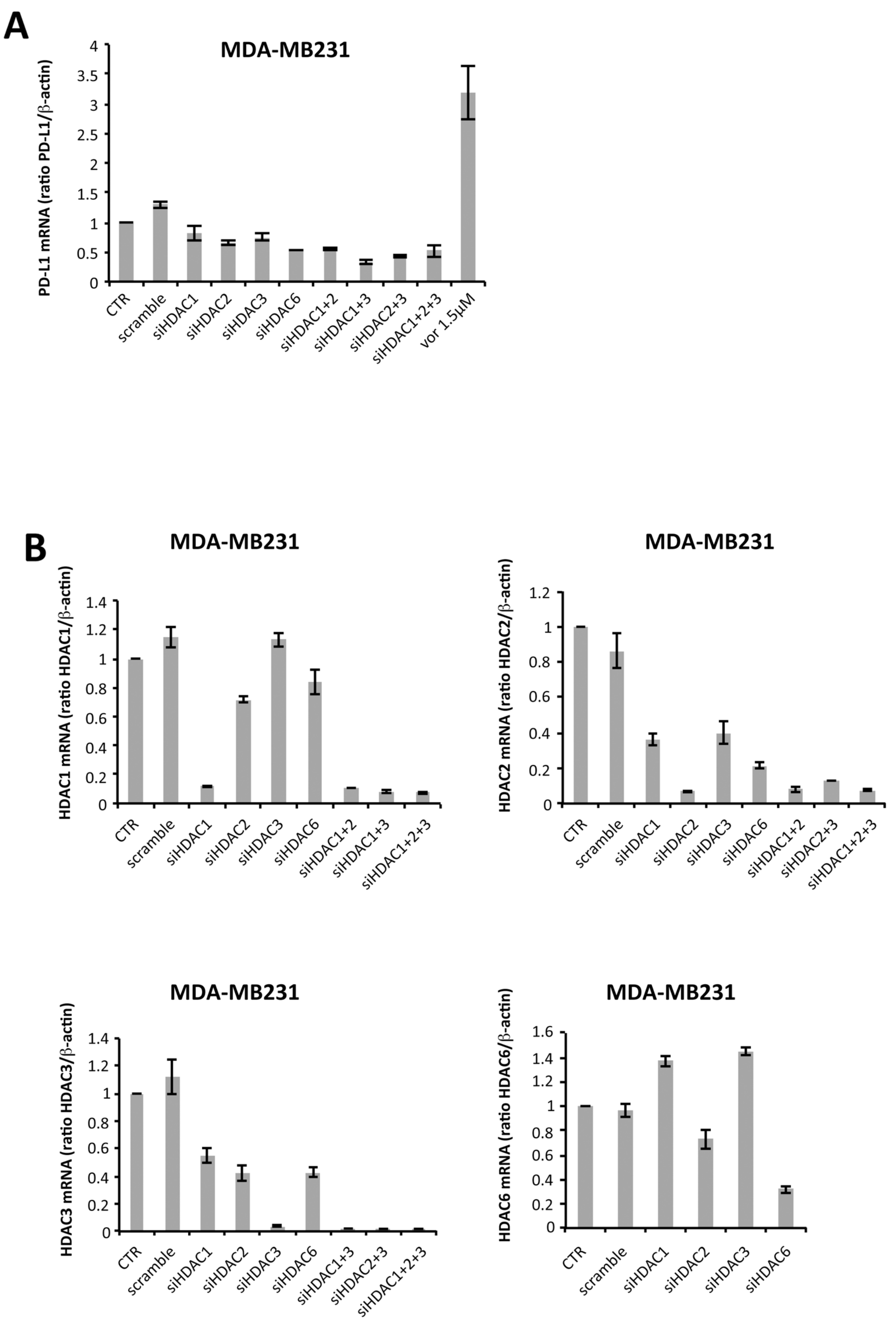

Supplementary Figure 3: PD-L1 up-regulation involves the inhibitions of multiple isoforms of HDACs. (A) MDA-MB231 cells were exposed to HDAC1, HDAC2, HDAC3, HDAC6 or scrambled (33 nM) siRNA for 24 hours. The expression level of PD-L1 mRNA was evaluated by qReal-Time PCR. **(B)** The efficiency of HDAC1, HDAC2, HDAC3 and HDAC6 knockdown was verified by qReal-Time PCR. β-actin was used as the housekeeping control gene to which the expression levels obtained by qReal-Time PCR were normalized. Data are presented as the mean ± SD.

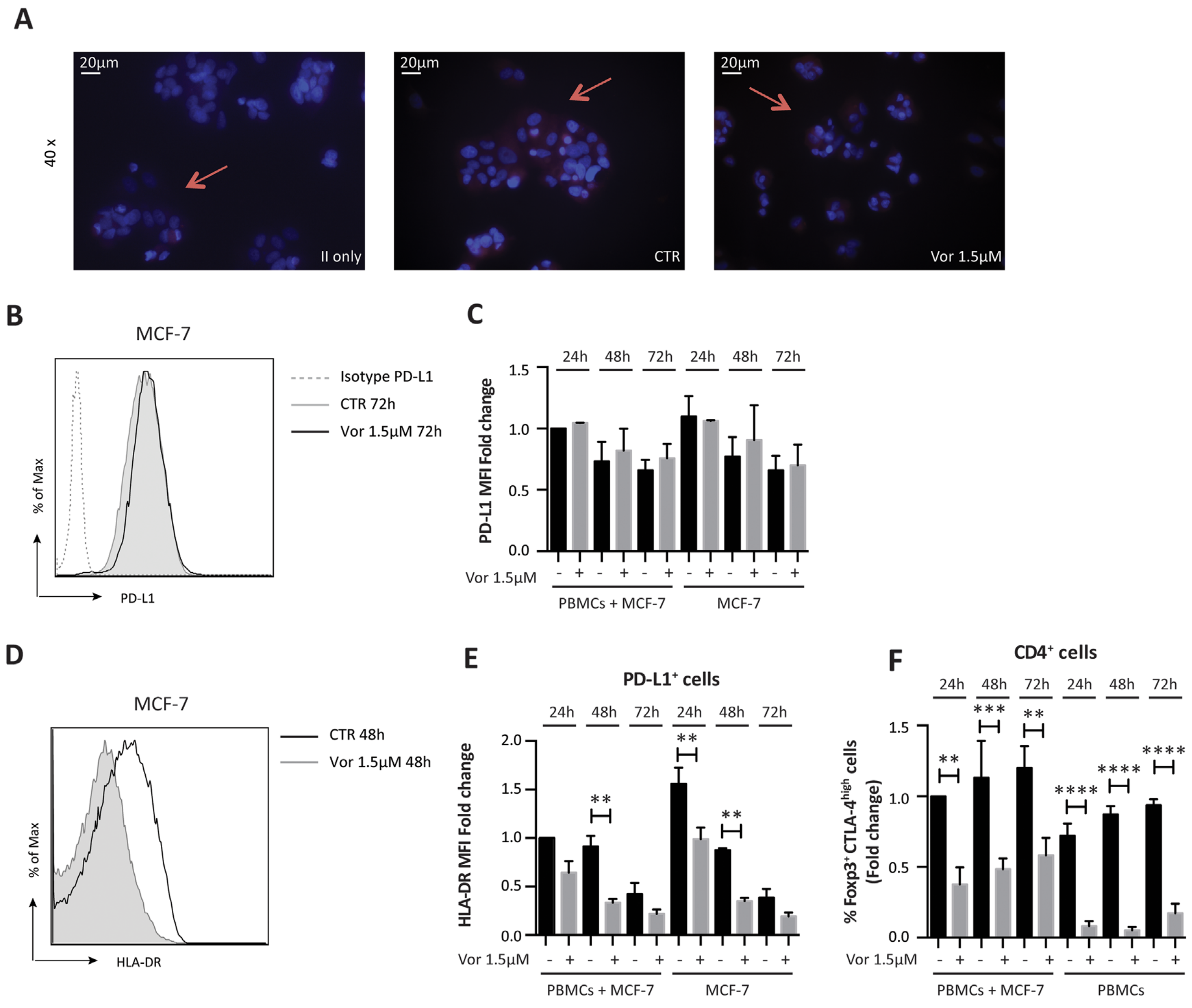

Supplementary Figure 4: Vorinostat effect on MCF-7 breast cancer cells and PBMCs in the co-culture system. **(A)** MCF-7 cells untreated or treated with vorinostat 1.5μM for 48 hours were fixed, stained for PD-L1 (red) and DAPI for nuclei (blue) and observed by microscope. Representative images show PD-L1^{+} cells with 40x magnification: first image shows the background signal with the secondary antibody alone, second and third images show PD-L1 expression in untreated and treated MCF-7 cells. Representative flow cytometric plot of PD-L1 **(B)** and HLA-DR **(D)** expression in MCF-7 cells with or without vorinostat treatment. Flow cytometric quantification of PD-L1 **(C)** and HLA-DR **(E)** expression in MCF-7 cells alone or co-cultured with PBMCs after 24, 48 and 72 hours of vorinostat treatment. **(F)** Flow cytometric quantification of CD4^{+} Foxp3^{+} CTLA-4^{high} T cells in PBMCs from healthy donors alone or in presence of MCF-7 cells after 24, 48 and 72 hours of vorinostat treatment. Data are presented as the mean ± SEM. Statistical significance is indicated by p-values as ** P ≤ 0.01; *** P ≤ 0.001; **** P ≤ 0.0001.

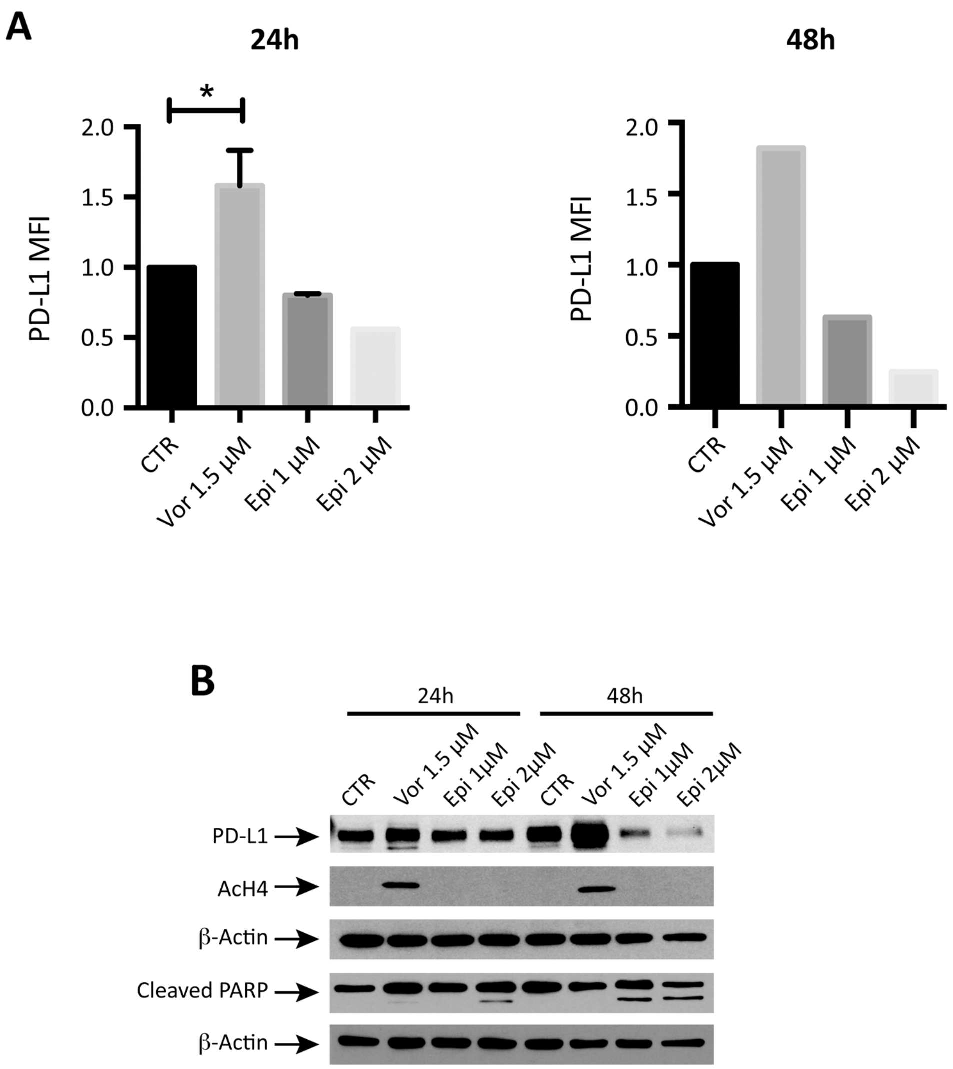

Supplementary Figure 5: PD-L1 up-regulation induced by vorinostat treatment is not related to an apoptotic effect. MDA-MB231 cells were treated or not with vorinostat (1.5µM) or epirubicin (1µM and 2µM) for 24 and 48 hours. Cells were then collected and PD-L1 expression was evaluated by flow cytometry and western blot. **(A)** Flow cytometric quantification of PD-L1. Data are presented as the mean ± SD. Statistical significance is indicated by p-values as * $P \leq 0.05$ and was determined by one-way ANOVA followed by Tukey's multiple comparisons test. **(B)** PD-L1, AcH4 and PARP protein expression was evaluated by western blot. PARP cleavage followed by epirubicin treatment was considered as positive control, indicating apoptotic effect. β-actin was used as protein loading control.

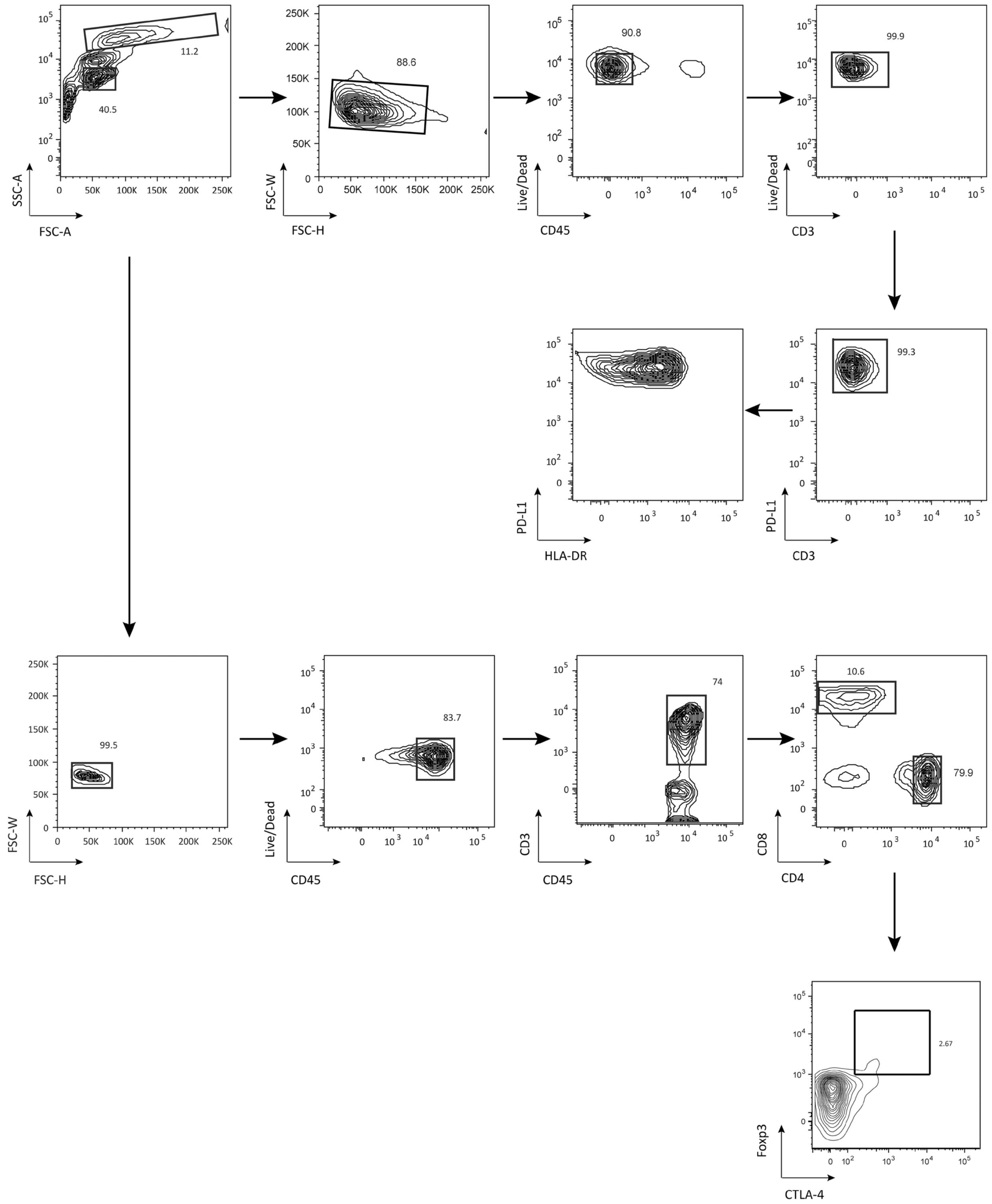

Supplementary Figure 6: Flow cytometry gating strategy. For isolation of CD4$^+$ Teff cells (live CD45$^+$CD3$^+$CD4$^+$Foxp3$^-$), CD4$^+$ Treg cells (live CD45$^+$CD3$^+$CD4$^+$Foxp3$^+$CTLA-4^{high}), cytotoxic CD8$^+$ T cells (live CD45$^+$CD3$^+$CD4$^-$CD8$^+$) and tumor cells (live CD45$^-$ CD3$^-$PD-L1$^+$).

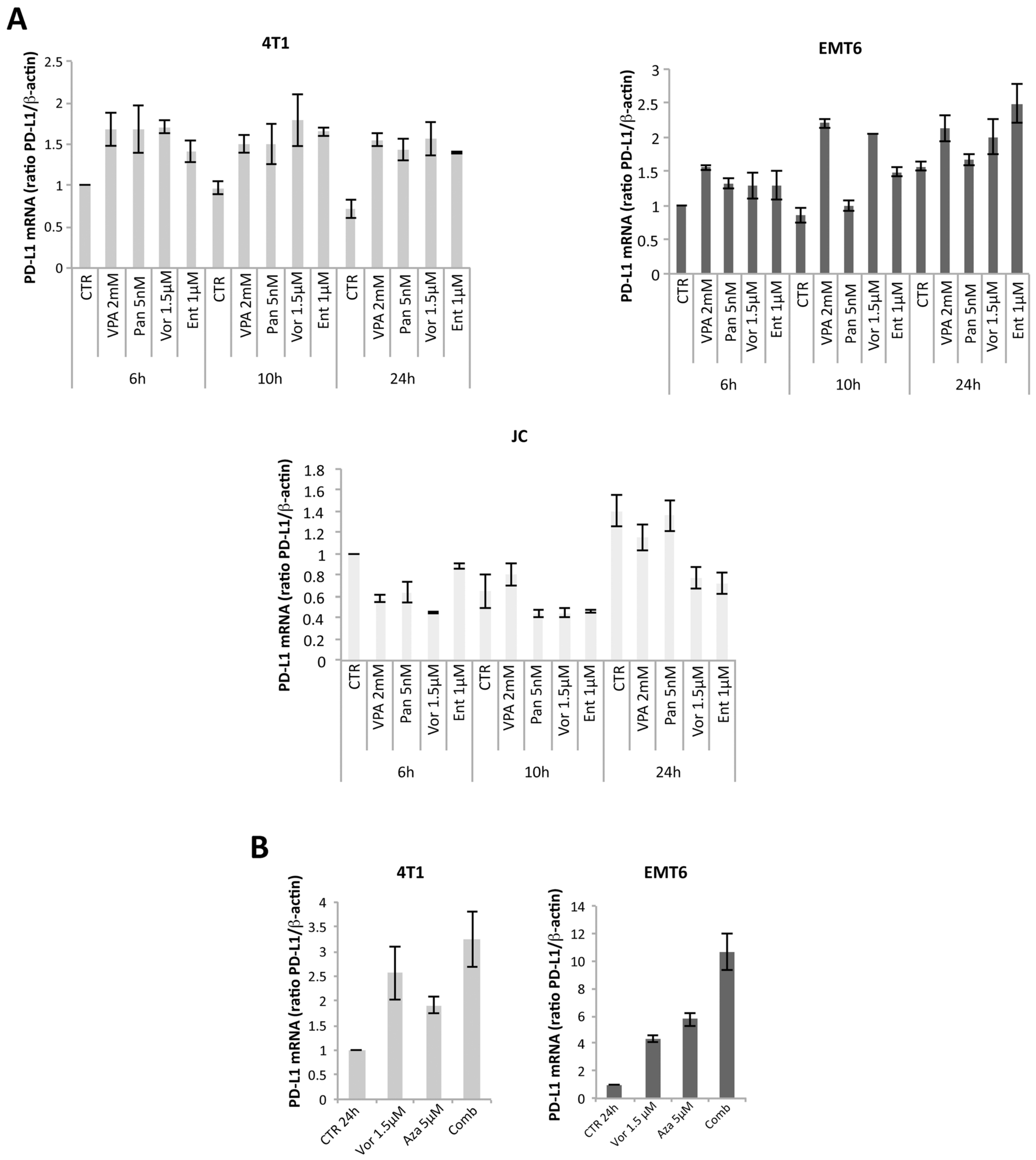

Supplementary Figure 7: HDACi induce PD-L1 expression in mouse breast cancer cell lines. (A) 4T1, EMT6 and JC mouse cell lines were untreated or treated with HDACi valproic acid (VPA), panobinostat (Pan), vorinostat (Vor) and entinostat (Ent) at the indicated concentrations for 6, 10 and 24 hours. **(B)** 4T1 and EMT6 cells were treated with vorinostat (Vor 1.5µM) and/or azacitidine (Aza 5µM) alone and in combination for 24 hours. PD-L1 mRNA expression was evaluated by qReal-Time PCR with β-actin used as the housekeeping control gene. Data are presented as the mean ± SD.

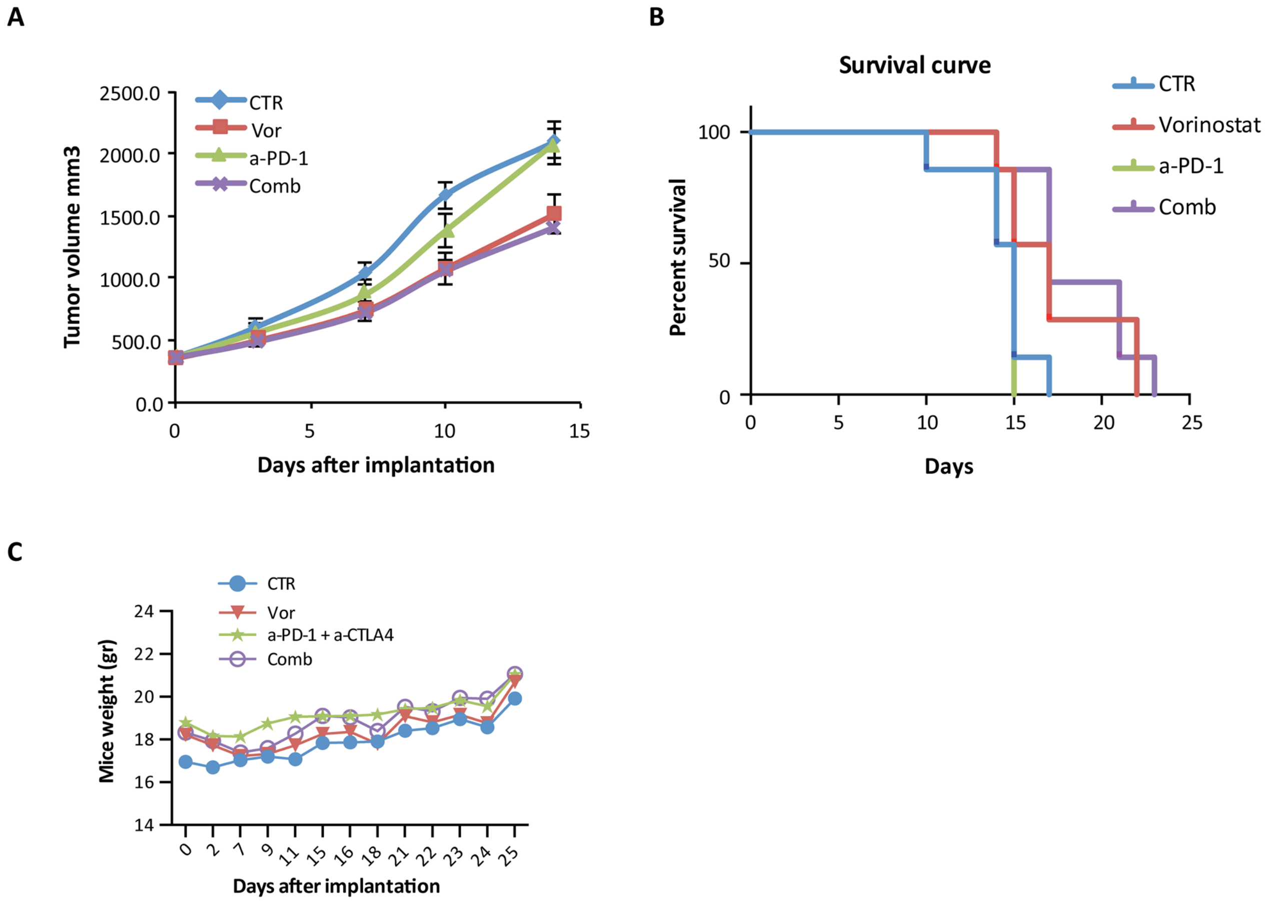

Supplementary Figure 8: Vorinostat in combination with anti-PD-1 blockade is not sufficient for *in vivo* synergistic anti-tumor effect. (A) 4T1 cells (5 x 10^6) were s.c. injected into BALB/C mice as described in the Materials and Methods. When established tumors reached a volume of 400 mm^3, mice were treated with vorinostat (50 mg/Kg i.p.), anti-PD-1 (10 mg/Kg i.p.) or both drugs in combination. Vorinostat was given 5 days/week, while anti-PD-1 was given on day 1, 3 and 5 with this schedule repeated for two weeks. (A) Relative tumor volume curves for 4T1 allograft. Mean ± SEM tumor volume measured at pre-specified time points (n = 8). **(B)** Effect of vorinostat and/or anti-PD1 treatment on the survival of 4T1 allograft mice. **(C)** Body weight measured three times/week during the three drugs combination study (vorinostat + anti-PD-1 + anti-CTLA-4).

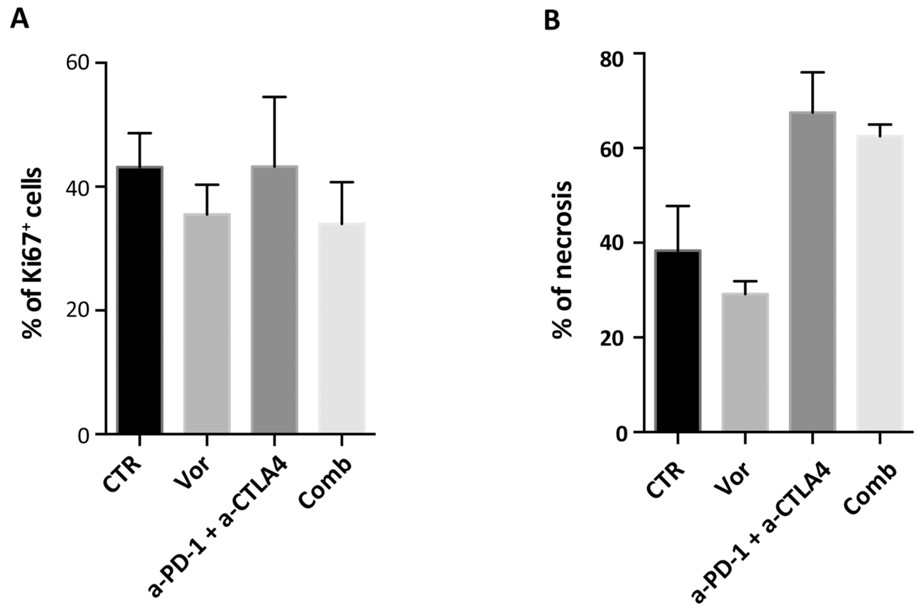

Supplementary Figure 9: Effect of vorinostat/immunotherapy treatment in 4T1 allograft tumors. Paraffin-embedded tissue was generated from each tumor for IHC. **(A)** The proliferative index was determined by immunohistochemical detection of Ki67 expression. Whole slide images of Ki67-immunostained tumor sections were scanned using an Aperio ScanScope XT whole slide scanner (Aperio) and visualized using ImageScope software (Leica). Ki67 proliferation index was scored and quantitated as the average percentage of Ki67$^+$ tumor cell nuclei per total tumor cell nuclei in 4 captured fields per sample using Immunoratio software. At least 200 cells were counted per field. **(B)** Percentage of tumor necrosis was determined based on evaluation of H&E-stained sections. Data are presented as the mean ± SEM.

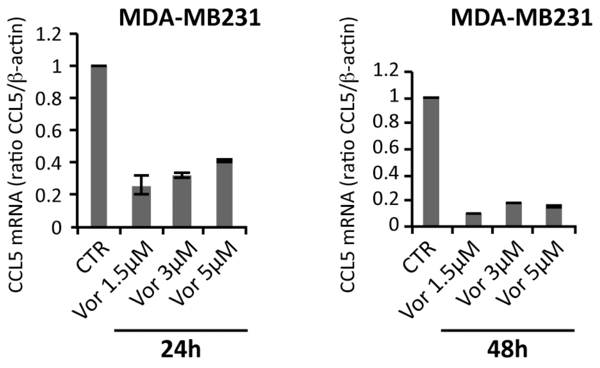

Supplementary Figure 10: Vorinostat reduces CCL5 mRNA expression in MDA-MB231 cells. MDA-MB231 cells were untreated or treated with increasing doses of vorinostat for 24 and 48 hours. CCL5 mRNA expression was evaluated at 24 and 48 hours after treatment by qReal-Time PCR. β-actin was used as the housekeeping control gene to which the expression levels obtained by qReal-Time PCR were normalized. Data are presented as the mean ± SD between triplicates.
